# Activity Models of Key GPCR Families in the Central Nervous System: A Tool for Many Purposes

**DOI:** 10.1021/acs.jcim.2c01531

**Published:** 2023-05-31

**Authors:** Shayma El-Atawneh, Amiram Goldblum

**Affiliations:** Molecular Modelling and Drug Design Lab, Institute for Drug Research and Fraunhofer Project Center for Drug Discovery and Delivery, Faculty of Medicine, The Hebrew University of Jerusalem, Jerusalem 91905, Israel

## Abstract

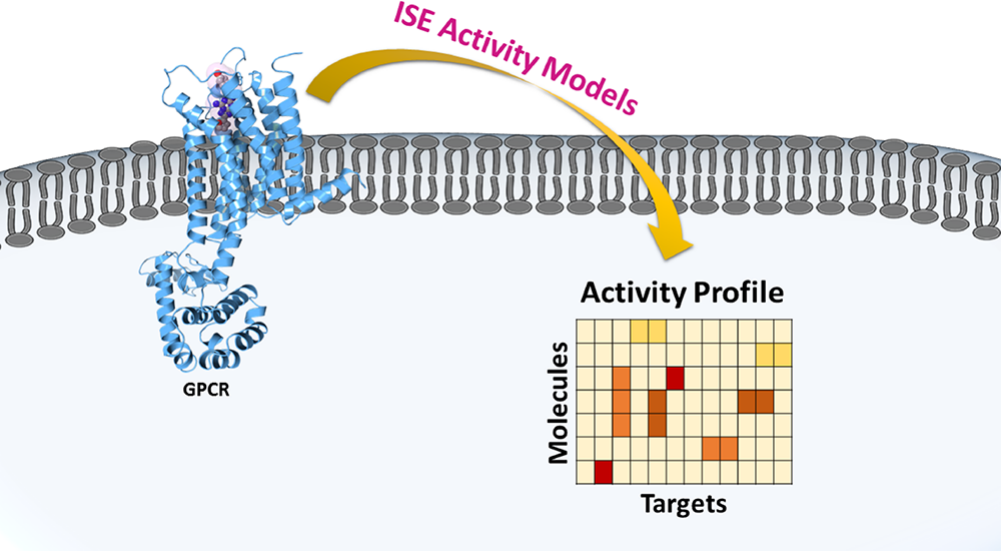

G protein-coupled receptors (GPCRs) are targets of many drugs, of which ∼25% are indicated for central nervous system (CNS) disorders. Drug promiscuity affects their efficacy and safety profiles. Predicting the polypharmacology profile of compounds against GPCRs can thus provide a basis for producing more precise therapeutics by considering the targets and the anti-targets in that family of closely related proteins. We provide a tool for predicting the polypharmacology of compounds within prominent GPCR families in the CNS: serotonin, dopamine, histamine, muscarinic, opioid, and cannabinoid receptors. Our in-house algorithm, “iterative stochastic elimination” (ISE), produces high-quality ligand-based models for agonism and antagonism at 31 GPCRs. The ISE models correctly predict 68% of CNS drug-GPCR interactions, while the “similarity ensemble approach” predicts only 33%. The activity models correctly predict 56% of reported activities of DrugBank molecules for these CNS receptors. We conclude that the combination of interactions and activity profiles generated by screening through our models form the basis for subsequent designing and discovering novel therapeutics, either single, multitargeting, or repurposed.

## Introduction

G protein-coupled receptors (GPCRs) are the most abundant transmembrane proteins in nearly all essential biological processes.^1^ Activation of GPCRs by different stimuli (photons, odorants, neurotransmitters, and hormones) initiates signaling through heterotrimeric G proteins and G protein-independent pathways by G protein-coupled receptor kinase (GRK)-mediated phosphorylation and arrestin coupling.^2^ GPCRs are classified based on evolutionary and sequence conservation.^3,4^ Drugs approved for conditions associated with specific GPCRs are promiscuous and interact on average with six to seven other GPCRs.^5^ Many more drugs targeting GPCRs are expected to be discovered as current therapeutics target only ∼25% of the potentially druggable human GPCRs.^6^

We focus on the class A GPCR family, which has a well-established druggability profile and is the largest and most diverse GPCR family (∼700 receptors). The class A family is widely distributed in the central nervous system (CNS) and is vital in regulating neuronal activity and behavior, ranging from motor control to learning, memory, and brain stimulation reward (Table 1). Given the wide range of their physiological functions, class A is the most targeted therapeutically compared to other GPCR classes, with over 500 approved drugs.^7^ Until 2017, 475 drugs were approved for the following GPCR families: 7% of drugs to opioid receptors, 11% to dopamine, 12% to serotonin (5-hydroxytryptamine, 5-HT), 12% to muscarinic acetylcholine (Ach), and 14% to histamine receptors.^8^

**Table 1:** Selected GPCR Families and Their Role in the CNS and CNS Disorders

GPCR family	effects on the CNS
serotonin receptors (5-HTR)	neuropsychiatric disorders, sleep–wake cycles, emesis, appetite, mood, memory, breathing, and cognition^13^
dopamine receptors	control of locomotion, cognition, emotion, learning and memory, motivation, reward, and neuroendocrine secretion^14^
muscarinic receptors	regulation of movement control, cognitive function, nociception, and body temperature, modulation of neuronal excitability, synaptic plasticity, and feedback regulation of Ach release^15,16^
histamine receptors	arousal, anxiety, activation of the sympathetic nervous system, the stress-related release of hormones from the pituitary and central aminergic neurotransmitters, antinociception, water retention, and suppression of eating^17,18^
opioid receptors	sedation, analgesia, euphoria, and respiratory depression^19^
cannabinoid receptors	disruption of short-term memory, cognitive impairments, discoordination, sleepiness, hypnotics, analgesics, antiemetics, antiasthmatics, neuroprotective, antiepileptics, eating disorders, and alcohol withdrawal^20^

The most targeted family by 253 CNS drugs (complete list in Table S1) is serotonin, with 58 drugs, followed by dopamine (46), histamine (33), muscarinic Ach (28), and opioid (25) (Table S2). Cannabinoid receptors (CBR) are currently targeted only by three approved CNS drugs, but we modeled this family because of their role in CNS disorders. They have become an extremely popular target in preclinical development for CNS disorders.^9^

There is a need for more effective, better tolerated, and safer therapeutic agents for CNS disorders because of (i) limited response (only ∼50% respond to the first treatment in depression) and high rates of relapse (∼30% entered remission), (ii) treatment resistance (failure of response despite target engagement), (iii) restricted symptom control, such as poor relief of negative symptoms and cognitive impairment, (iv) side effects including motor, sleepiness, and sexual dysfunction, and (v) long-term safety issues such as cardiovascular, metabolic dysregulation, and weight gain.^10^ Clinical development for treating CNS disorders has one of the lowest success rates in the pharmaceutical industry.^11^ For example, between 2006 and 2013, more than 200 anti-Alzheimer’s disease drug candidates failed clinical trials.^12^

The general similarity of seven-transmembrane (7TM) helices of GPCRs, including homologous residues within the orthosteric binding sites in the A family,^21^ restricts the ability to develop selective drugs for GPCRs. For example, cholinergic side effects of M1 agonists are caused by the activation of M2 and M3 receptors, which have highly conserved orthosteric acetylcholine binding sites compared to the M1 receptor.^22,23^

Polypharmacological compounds (which affect more than one receptor) may demonstrate better clinical efficacy,^24,25^ such as atypical antipsychotics (clozapine and aripiprazole) interacting with numerous GPCRs.^26^ Such multiple targets are merely accidental outcomes, not being designed or planned. In most cases, hitting unwanted targets produces adverse reactions (“side effects”). A noticeable case is the unwanted activation of the 5-hydroxytryptamine 2B (5-HT2B) receptor, expressed on heart valve leaflets, leading to proliferative valvular heart disease.^27^ The design of effective multitargeting therapeutics has to consider both targets and anti-targets (those that should be avoided). The general similarity also suggests repurposing opportunities, i.e., new targets identified for drugs approved previously for other GPCR targets.^28^

Computational methods have been used to address these issues with some success but cover a small number of targets.^29^ Several docking strategies predict the affinity of drugs to their GPCR targets and off-targets^30−33^ and are employed to design candidates for binding to GPCRs.^34,35^ Docking has a few general limitations due to the simplified scoring functions^36,37^ and lack of structural flexibility.^38^ Pharmacophore approaches have been used extensively in virtual screening (VS), de novo design, lead optimization, and multitargeting drug design.^39−41^ Other ligand-based methods were used to discover ligands for various GPCRs.^42−48^ Among those methods, our in-house algorithm, “iterative stochastic elimination” (ISE),^49^ was recently employed to discover novel cannabinoid-1 (CB1) receptor antagonists.^50^ There are many other approaches for predicting GPCR-molecule interactions.^51^

Most GPCR structures still need to be elucidated because detailed structure information requires large-scale expression and purification due to their highly dynamic nature.^52,53^ Structures are currently reported for ∼120 members of class A GPCRs.^54^ This gap may be partially filled by computational modeling^56−59^ and by improvements in the quality of cryo-EM of GPCR structures.^55^ On the other hand, much data about ligand binding and action has been accumulated, including GPCR activation and blocking of GPCR activity. Therefore, in the GPCR field, ligand-based methods may be a good alternative to structure-based ones.

This paper describes the construction of activity models for 31 GPCRs involved in CNS disorders by the ISE algorithm. Our ISE is a unique and unprecedented generic classification algorithm for finding reasonable solutions to highly complex combinatorial problems.^56^ For drug classifications,^50,57−60^ a learning set comprises molecules known to have an activity (i.e., agonists, antagonists, or inhibitors) on a specific target, diluted by a considerable number (100- or 1000-fold) of assumed inactive molecules. The algorithm randomly picks five property filters out of ∼200 physicochemical properties of each molecule to distinguish between the active (“positive”) and the inactive compounds. Statistical criteria evaluate the ability of each “property filter” to classify. After picking many such filters, it is possible to determine which properties are consistently associated with the worst statistics, and those are eliminated. The process continues in iterations until a smaller number of combinations enable quickly picking all the remaining possible filters of five properties and sorting them according to the statistic criteria (see the Methods section). A model consists of many filters that provide the best classifications. Such ISE models are our basis for virtual screening.

## Results

### ISE Activity Models

**Figure 1:**
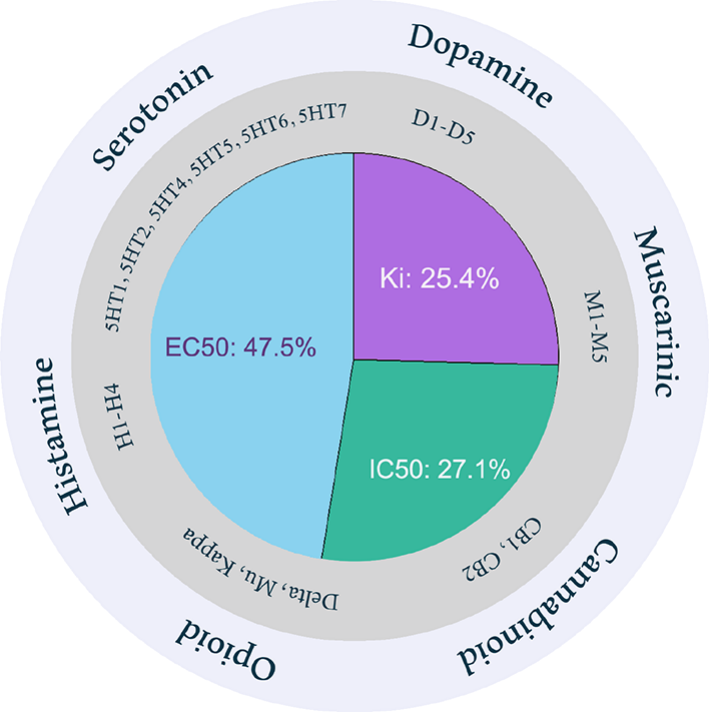
Fifty-nine activity models with the best performance were chosen for further studies; 28 models of agonist activity (47% with EC_50_) and 31 models of antagonist activity (53% with *K_i_* and IC_50_ values).

The performance of the ISE models for agonists and antagonists of 31 receptors from six different families is presented in the Supporting Information, Table S3. Agonists are based on EC_50_ values, and antagonists are based on IC_50_ or *K_i_* values (Figure 1). Due to the lack of enough data in ChEMBL,^61^ we could not construct models for dopamine D5 agonism (EC_50_), 5-HT1E agonism (EC_50_), and 5-HT5A agonism (EC_50_). Thus, we present 59 models: 28 agonist and 31 antagonist activity models.

### Statistics of the ISE Models

Most models have a relatively large number of active compounds, enabling 5-fold cross-validation.^62^ Two models with few reported active compounds (below 20 molecules) were constructed with LOO validation,^63^ the agonist models of 5-HT7 and H1. Each model comprises the best filters for distinguishing between active and assumed inactive molecules. The Matthews correlation coefficient (MCC)^64^ ranks the filters, with the top filter having the best MCC closest to +1. The average MCC value of the top filter in each fold is presented in Table S3. The agonist models’ mean MCC ranges between 0.68 and 0.99 with an average of 0.84; for the antagonist models, it ranges between 0.55 and 0.95 and an average of 0.81 (Table S3). The worst mean MCC is for the CB2 receptor (CB2R) in both the agonist (0.68) and antagonist (0.55) models. The best MCC values are for 5-HT7 agonist (0.99) and M3 antagonist (0.95) models. The average MCC of all models is 0.83.

The area under the ROC curve (AUC) was calculated for each model’s test set from all five folds. The agonist models range between 0.57 and 0.99; the kappa receptor produced the best model (Table S3). All models have an AUC greater than 0.84, except for 5-HT7 (0.57). For the antagonist models, the AUC ranges between 0.85 and 0.99; the best model is 5-HT1D (0.99), and the lowest is the CB2R (0.85). The average AUC of all 59 models is 0.94. The TNR (specificity) is high for all models (>0.9), and the TPR or sensitivity is high for almost all models. TPR ranges between 0.47 and 0.84 for the agonist models, except for the 5-HT7 model with the lowest value of 0.1 (while its mean MCC of five filters is 0.99). We comment on the difference between the MCC and TPR in the Discussion section. The antagonist models’ TPR ranges between 0.46 and 0.9; the best model is for M3 antagonists, while two models have low values: the CB2 and 5-HT2B models (0.04 and 0.34, respectively). In general, the reported statistic values indicate good performance for all models.

### Learning Set Diversity and Statistical Evaluation

We compared the three models with top performance (based on the MCC, AUC, and enrichment factor (EF)) to the three least performers to find out if active set diversity affects the quality of the models. Average Tanimoto coefficients (Tc of the active molecules, of the top 100 of the screened molecules vs the active compounds, and the comparison within the top screened molecules) are shown in Table 2. The best three models have slightly higher average Tanimoto coefficient than the less performing ones, but all six have high diversity sets, with Tc < 0.7.

**Table 2:** Mean MCC, AUC, and EF for the Best Three Models (Top Three) and Least Performing (Bottom Three)^a^

	statistic performance			average Tanimoto coefficient (Tc)		
model	mean MCC	AUC	EF^b^	active compounds of the model	top 100 screened molecules vs active compounds	top 100 screened molecules: internal Tc
5-HT6 agonist	0.96	0.99	146	0.48	0.37	0.39
D1 agonist	0.92	0.98	146	0.44	0.34	0.38
M3 antagonist	0.95	0.99	135	0.50	0.34	0.38
M4 agonist	0.68	0.85	12	0.31	0.22	0.27
5-HT2B antagonist	0.65	0.92	35	0.35	0.32	0.37
CB2 antagonist	0.55	0.85	71	0.38	0.38	0.51

a The average Tc is presented for each model for the active molecules used to build each model and the top 100 scored molecules from screening the ZINC drug-like database. Matthews correlation coefficient (MCC), area under the ROC curve (AUC), enrichment factor (EF), and Tanimoto coefficient (Tc).

b Values above an index of 0.7.

Tanimoto values among the top-scored molecules from screening the ZINC drug-like database (DB) (17,900,742 molecules)^65^ were examined for the effect of learning sets’ diversity on VS results. The M4 agonist model has a lower average Tc when comparing the top 100 screened molecules to the active compounds used to build the model and among those 100 screened molecules. The CB2 antagonist model has the highest average Tc within the top 100 candidates compared to the other five models.

Does the choice of different random sets affect the performance of our models? To answer that question, we picked different random sets for the best and worst ISE models. As shown in Table 3, different random sets do not affect the performance of the 5-HT6 agonist and CB2 antagonist models.

**Table 3:** Performance of 5-HT6 Agonist and CB2 Antagonist Models with Different Random Sets^a^

model	random set	mean MCC	AUC	EF^b^	TPR^b^	TNR^b^
5-HT6 agonist (110 active molecules + 110,000 randoms)	1	0.96	0.99	146	0.9	0.99
	2	0.95	0.96	167	0.9	0.99
	3	0.95	0.96	104	0.91	0.99
CB2 antagonist (689 active molecules + 70,000 randoms)	1	0.55	0.85	71	0.04	0.99
	2	0.58	0.84	92	0.16	0.99
	3	0.57	0.83	77	0.34	0.99

a Matthews correlation coefficient (MCC), area under the ROC curve (AUC), enrichment factor (EF), true positive rate (TPR), and true negative rate (TNR).

b Values above an index of 0.7.

### Features Distinguishing Active Compounds from the Rest of the World

The filters that distinguish active compounds from others are constructed from physicochemical properties. The filters generated in each model vary in number and composition; however, all contain five ranges of different descriptors (Table S3).

The distribution of descriptor families (“types”) in the agonist and antagonist models is shown in Table 4, and a more detailed distribution per receptor is presented in Figures S1–S12. The dominant descriptor type all over the agonist and antagonist models is the “partial charge” type followed by “adjacency and distance matrix” and “atom and bond count” descriptors, while the “Kier and Hall connectivity” descriptors have a minor role. No descriptors were more favorable in one receptor family than others (Figures S1–S12). It is important to note that there are no constraints to picking only one descriptor from a family (descriptor type), so in principle, all descriptors in a single filter could be from a single family of descriptors.

**Table 4:** Average Occurrence (%) of the Descriptors’ Family for the Agonist and Antagonist Models

descriptor type	partial charge	atom and bond counts	pharmacophore feature	physical properties	subdivided surface areas	adjacency and distance matrix	Kier and Hall connectivity
agonist models	31	16	11	6	13	22	1
antagonist models	31	15	12	5	10	26	1

### Screening the DrugBank for External Validations

We screened 7130 drugs (downloaded from DrugBank^66^) by 59 activity models. Among those 7130, there are 1212 reported activities for only 361 drugs in three databases (DrugBank, ChEMBL, and TTD, see the Methods section Drug-Protein Matrices; Table S2). Our 59 models correctly predicted 684 activities (assuming any positive index as correct) out of the reported 1212, which amounts to a 56% success (Figure S13).

To validate these results, we performed Y-randomization: the reported activity matrix (of “1”s and “0”s) was randomly assigned while keeping the same number of activities (1212) and repeating that assignment 1000 times (Figure S13). By the same criterion (any positive index is a correct prediction), the average rate of randomization success is 5% (± 0.66%) compared to 56%.

Out of the 7130 drugs screened by our models, we predict 22,671 agonist and antagonist activities (index >0.0) for 2823 drugs. No GPCR activities were found for the rest, 4307 drugs. As the number of predicted activities is ∼8 times larger than that of drugs (2823), many drugs must be characterized as “polypharmacological active” according to our predictions. This polypharmacological profile may explain these drugs’ side effects or mechanisms of action.

**Figure 2:**
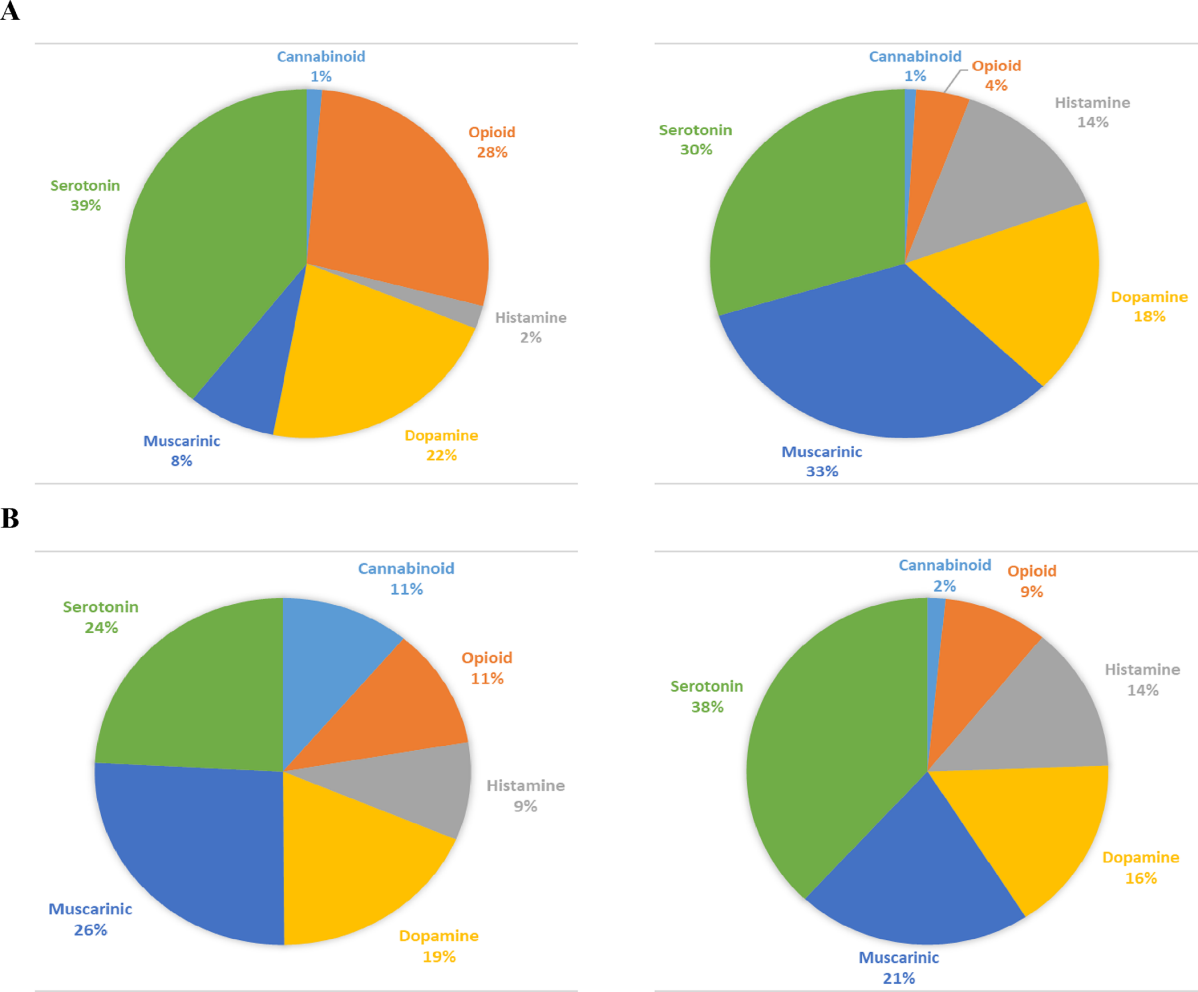
Agonist and antagonist activities per GPCR family. (A) Reported agonist (left) and antagonist (right) activities (370 and 842, respectively). (B) The predicted activities by the ISE models were 10,895 agonist (left) and 11,776 antagonist activities (right).

Among the 1212 known activities of the 361 drugs, there are more reported antagonist activities (842) than agonist activities (370) (Figure 2A). However, our predictions for 2823 drugs suggest similar-sized agonist (10,895) and antagonist (11,776) activities (Figure 2B). The differences among the 361 drugs probably reflect greater therapeutic demand for antagonists. Of the reported activities, there is a larger percent of opioid, serotonin, and dopamine agonists than antagonists, a larger percentage of muscarinic and histamine antagonists than agonists, and a similar percentage of cannabinoid agonists and antagonists. Among the predicted results, there is a larger percentage of serotonin and histamine antagonists than agonists and a greater percentage of agonists than antagonists for the other families.

To focus only on drugs directed to CNS disorders, we find that 112 out of 253 CNS drugs (Table S1) have 565 reported activities with the 31 GPCRs. Our models correctly identified 312 activities (55%). We also found 2502 new activities of the 253 CNS drugs. Figure S14 presents the heatmap for the screening results of the 253 CNS drugs through the 59 activity models.

### Comparison of Algorithms for Predicting CNS Drug Targets: ISE vs SEA

We compared our predictions of protein targets of drugs to another method that provides information on binding to proteins by a different approach. The similarity ensemble approach (SEA) has been used in some publications^67−69^ and can be compared to our ISE for the 31 GPCRs. However, SEA suggests only targets but not activities, so we compare the performance of ISE models to SEA only for target prediction.

The total number of literature-reported interactions is 529 (112 drugs out of the 253 CNS drugs), and our models achieve 68% correct predictions (362 interactions; Table 5, above an index of 0.0). For SEA, we applied an equivalent acceptable *P*-value cutoff of 10^–5^ as being correct (excluding results with a maximum Tc = 1). The SEA predicts 177 correct interactions out of the 529 reported ones, a 33% success, compared to 68% by ISE.

**Table 5:** Reported and Predicted Number and Average Interactions per Drug/Receptor for the CNS Drugs^a^

		# interactions per drug	Avg. interactions per drug	# interactions per receptor	Avg. interactions per receptor	total number of interactions
CNS drugs	reported interactions (112 drugs)	1–20	4.7 ± 4	1–45	17 ± 11	529
	predicted interactions (165 drugs)	1–24	13.3 ± 7	11–127	70.7 ± 34	2191

a The reported data are from DrugBank, ChEMBL, and TTD. Predicted interactions are for positive indexes of ISE. #, number; Avg, average.

**Figure 3:**
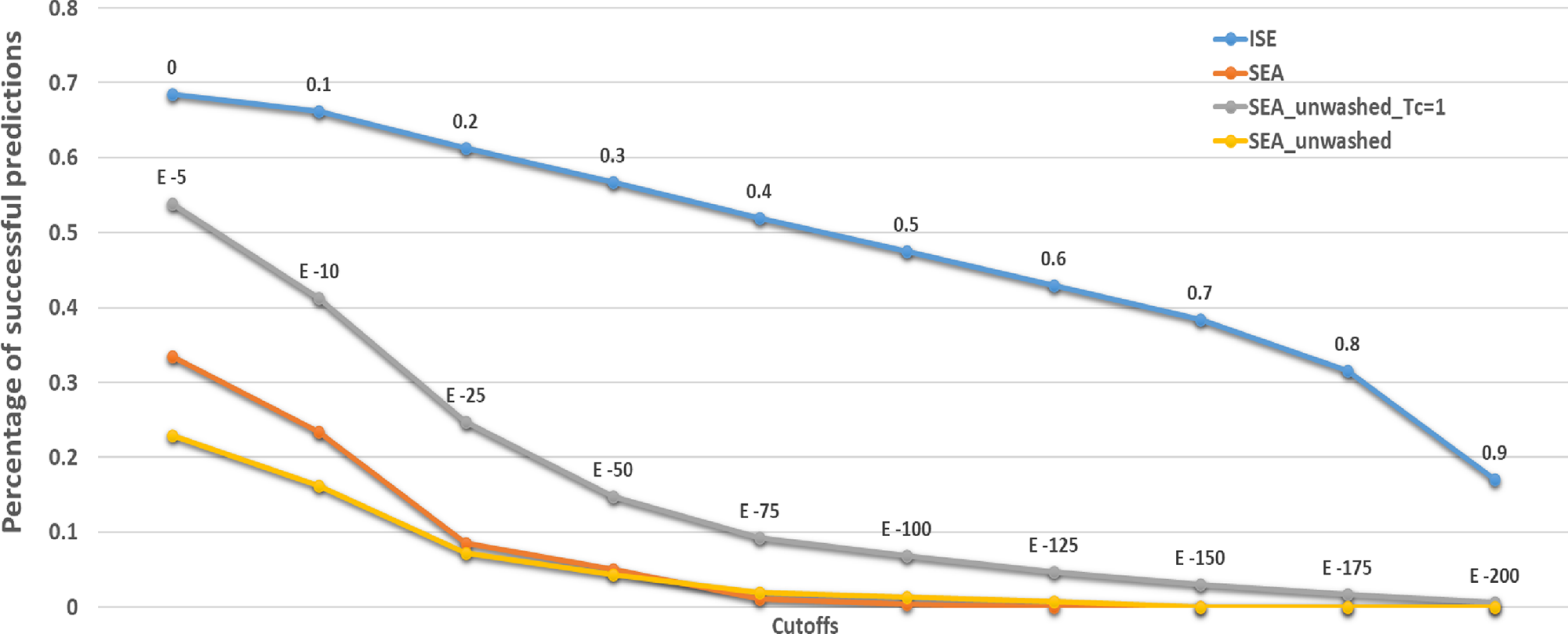
Percentage of successful predictions at different cutoffs for ISE (blue) and SEA (orange) with washed SMILES. Two other lines present the results of SEA with unwashed smiles: the gray line includes results with Tanimoto similarity = 1, while excluding Tanimoto = 1 results is shown by the yellow line. Both algorithms with more stringent cutoffs predict fewer interactions.

A reviewer suggests that the difference between these results is due to using the “washed” SMILES presentation (rejecting counterions and presenting the charged state of weak acids and bases by protonation or deprotonation) as input to SEA. Using “unwashed” SMILES, SEA finds 285 correct results (54%). However, for the predictions by SEA, 164 of those 285 results are comparisons of a drug to itself, being a ligand included in the learning set of ligands for that target (and expressed with a maximum Tanimoto value of 1). Removing such self-comparisons reduces the success of the SEA to 23% (Figure 3).

Using “washed SMILES” is a better presentation of the activity of these drugs at their receptors due to the vital role of protonated and deprotonated (i.e., charged) molecular species for binding to GPCRs.^70−72^ More than 95% of the 361 drugs we examined have a charged amine function or a quaternary nitrogen one.

Using more stringent values (for ISE indexes and *P*-values, see Figure 3) leads to fewer predicted interactions by both methods. Figure S15 presents the percentage of successful predictions per receptor in each method. ISE identifies targets better than the SEA for all receptors except for 5-HT7, D1, and the opioid delta and kappa receptors.

### Virtual Screening by GPCR Models for Suggesting Potential Candidates

The GPCR models are useful for discovering potential candidates from massive chemical databases by performing VS and scoring. Each model scores every molecule, and top-scored molecules may be further processed depending on the specific task—hitting a single target or multiple targets.

**Figure 4:**
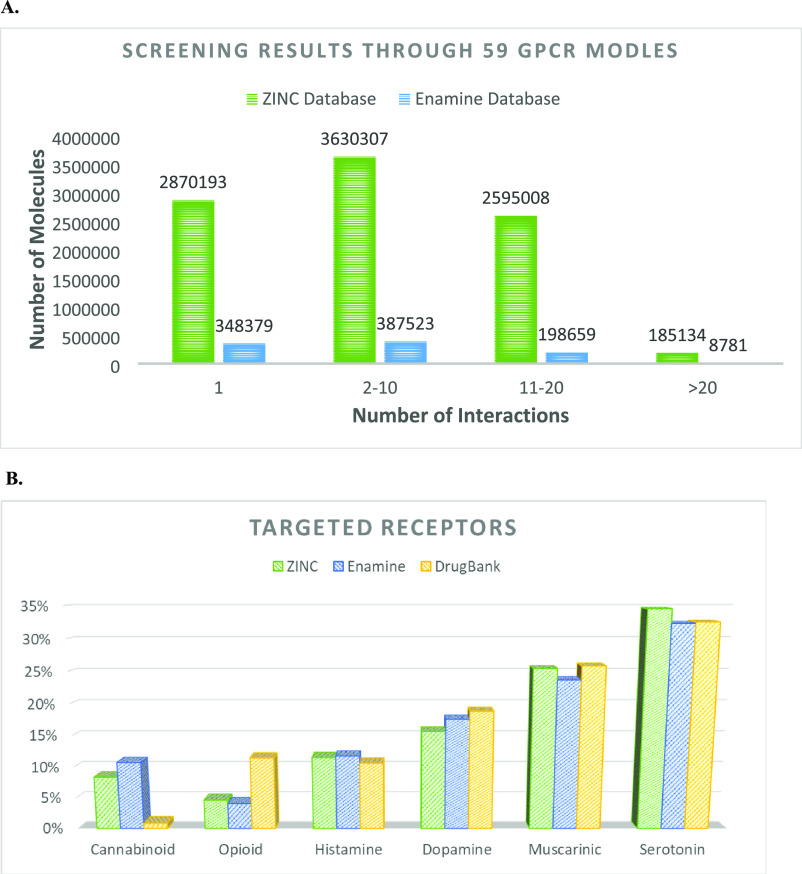
Virtual screening of the ZINC and Enamine databases. (A) Number of interactions per molecule with the 31 GPCRs by screening the ZINC DB (green) and the Enamine DB (blue) molecules with a positive index >0. (B) Distribution of the targeted GPCRs as reported by the DrugBank DB (yellow) and by screening the ZINC (green) and Enamine (blue) databases through the ISE models for molecules with an index score above zero.

Screening by the 59 GPCR activity models, we found that extensive groups of molecules are potential candidates as agonists or antagonists for our GPCR set. Screening the ZINC drug-like DB (17,900,742 molecules)^65^ yields 9,280,642 molecules (52% of ZINC) with one or more targets, with any positive index >0. These molecules have 1–30 interactions per molecule, with an average of 6.7 ± 6.1 interactions. Screening of the Enamine DB (2,159,632 molecules)^73^ yields 943,342 molecules (43% of this DB) with any positive score, with 1–28 interactions per molecule and an average of 5.5 ± 5.6 (Figure 4A).

The distribution of the targeted GPCRs in both ZINC and Enamine VS compared to the reported interactions in the DrugBank DB (v. 4.5)^66^ is shown in Figure 4B (1165 reported drug-GPCR interactions for 361 drugs out of the 7130 DrugBank DB and the 31 GPCRs, see Drug-Protein Matrices in the Methods section). The percentage of predicted interactions is almost similar between the two databases and the known reported drugs for the GPCR families. The differences are with opioids, with more known drugs than predicted, and with cannabinoids, where many more are predicted than are known.

## Discussion

In this paper, we present the construction of activity models for the main GPCRs, which may serve as a basis for learning and discovering drug candidates. Our models are based on reported results for ligand activities at these receptors: 28 agonist models (with reported EC_50_ values) and 31 antagonist models, of which 16 are based on IC_50_ and 15 on *K_i_* values. There is no notable difference in statistical performance between the agonist and the antagonist models (Table S3). The average mean MCC over agonist models is 0.84; for antagonists, they are 0.8 for IC_50_ and 0.82 for *K_i_*. The average AUCs for the antagonist models are slightly larger, 0.95 and 0.96 (IC_50_ and *K_i_*, respectively), compared to 0.93 for agonist models. The minor differences between the IC_50_ and *K_i_* models suggest that mixing their values for modeling the group of antagonists may be useful, particularly if the data size of one of them is small.

### Comparing Our Results to Others

A direct comparison of our results to others is not possible because other algorithms focus on different issues. A recent paper^74^ employed random forest (RF) models in two steps: first, the authors distinguished between GPCR-targeted and GPCR-nontargeted drugs (their T-model, with AUC = 0.79 and MCC = 0.44). In the second step (their A-model, with AUC = 0.82 and MCC = 0.51), they collected data from 134 FDA-approved GPCR-targeted drugs but used only 63 of those to determine only if they were agonists or antagonists without relating to any specific GPCR subfamily. Out of those 63, 44 were correctly assigned as either agonists or antagonists (their Table 2(74)), amounting to 70% correct assignment. However, they assign GPCR activity (A-model) to 11 drugs predicted as GPCR-nontargeted (T-model). Their T-model and A-model have worse AUC and MCC values than our worst results (Table 2). Our results are thus better than these recent results and are much more elaborate because we modeled specific receptors and, by that, supplied the possibility to relate directly to each of a major set of receptors.

Another paper employed multiple supervised ML algorithms (random forest, extreme gradient boosting (XGBoost), SVM, multi-layer perceptron (MLP), and deep neural networks (DNN)) to classify known GPCR ligands according to their GPCR targets (by grouping the GPCR subtypes into a single type).^75^ The average MCC of the five algorithms ranges from 81 to 92%.

The most relevant comparison is to SEA. We compare to SEA based on predicting interactions, not activities. On that equal basis and limited to our 31 receptors, we have shown that our success rate for target prediction of drugs rises to 68%, compared to 33% success for SEA when screening the same CNS drugs. This is clearly a substantial difference in predictive power between the two algorithms.

For activities, we present detailed results for ∼30 agonist and ∼30 antagonist activity models of the main CNS receptors. We correctly predicted 684 activities from the reported 1212 (56% success). The “false positive” activities that our models predict may be used to explain side effects and mechanisms of actions of unknown yet or unreported interactions.

### Which Criteria Reflect the Quality of Predictions?

We use several statistical measures of success, but the ultimate measure is that of experimental results. Our measures of success, such as the MCC, AUC, EF, sensitivity (TPR), and the ratio of true positives to false positives (TP/FP) above a specific scoring index, should all be characterized as “postdiction” as they all emerge from a learning set and not from predictions. For testing our predictions, we use the model’s TP/FP values, which vary with the scores (“indexes”, see the Methods section, eq 5) and look for the top TP/FP values at higher indexes to use as the index cutoff while performing VS. TP/FP reflects the chance to discover novel active compounds by sending *N* × (TP + FP) molecules for testing (*N* ≥ 1). If we wish to find candidates to hit more than a single target, then the overall chance should be multiplied by the chances for the different targets. Thus, the chance to discover active molecules that are multitargeting is considerably smaller than the chance to discover candidates for a single target.

Quality measures are confusing in some cases, such as 5-HT7 with an MCC of 0.99 and a TPR of 0.1. The MCC requires four numbers (eq 1), while TPR is composed of only two (eq 2). TPR (TPR = TP/(TP + FN)) may be small if most true positives are identified as false negatives. A large MCC requires that TP × TN ≫ FP × FN. A large TN (making FP very small) can still provide a large MCC value if FN is large. In such a case, where the number of active molecules for the 5-HT7 agonist model is small (11 molecules) compared to the selected randoms (11,000 molecules), the MCC is well known as the best measure of success in such unbalanced classifications.^76−78^

### Diversity Did Not Produce Better Ligand-Based GPCR Models

The more diverse a set of active molecules, the more diverse the decoys, and the more difficult it should be to distinguish a highly diverse set of active compounds from random molecules picked by the “applicability domain” principle. Therefore, models built from highly diverse sets are expected to be of lower quality, while the less diverse sets are more easily distinguished from the rest of the “chemistry space”. Our top models (upper three in Table 2) have an average Tc of ∼0.47, and the least three performers have a Tc of ∼0.35, just a slight difference. However, all models have good diversity (average Tc of less than 0.7).

Thus, for the top three and the lower three models, the significant differences between their MCC and EF values are not reflected in the minor differences in the average Tc. It is also interesting to note that AUC differences do not follow those of the MCC and EF. The original diversity of the learning sets is “repeated” in the diversity of the screening results. The most diverse set of the M4 agonist model (average Tc = 0.31) yields the most diverse top 100 ranked molecules (average Tc = 0.27), but we cannot claim similarly for the less diverse M3 antagonist model (average Tc = 0.5). The top 100 screened molecules differ from the active molecules used to build each of the six models. Identifying active hits structurally different from the learning sets is a major result of using physicochemical properties rather than structural elements for applying ISE.^50,59,79,80^

### Selectivity of Action Is Linked to Physicochemical Properties

Our ISE algorithm produces models from filters that include physicochemical property ranges that can best distinguish between classes of molecules. Thousands of molecular descriptors may be calculated using different types of software.^81^ Out of those, we focus on ∼200 2D physicochemical properties calculated by MOE.^82^ We find descriptor families that appear more than others among the top filters of most of our GPCR activity models. The percentage appearance of some of the dominant 2D descriptor families is similar in the agonist and antagonist models (average results are given in Table 4). However, focusing on individual receptors, there are differences in the prevalence of descriptor families between models, as shown in Figures S1–S12.

Figures S1–S12 provide an enormous wealth of information that should help, if appropriately analyzed, to detect many commonalities and differences between these receptors. Such in-depth analysis of each receptor and its preferences for a specific descriptor family and specific properties should be the subject of further papers.

One of the prominent families of descriptors in both agonist and antagonist models is the partial charge family (Data S1). Those descriptors depend on the atomic VdW surface and the partial charge of each atom of a chemical structure. MOE uses two methods to calculate the partial charges: the “partial equalization of orbital electronegativity” (PEOE) method^83^ and Q descriptors that use the partial charges stored with each structure in the database. High occurrences of that family of descriptors (∼50%) are found in 5-HT2A/5-HT7/CB2/H2 antagonist and H1/M1 agonist models. Two models (out of 59) are not affected by charge issues: kappa and mu agonist models. Investigation of the class A GPCR ligand-binding pocket in the 7TM region indicates multiple topologically equivalent residues that form a consensus ligand binding network explaining cross-reactivity and polypharmacology.^84^ These GPCRs share the same distribution of amino acid classes among binding site residues. Half of the amino acids are aliphatic, ∼15% are aromatic, ∼20% are polar, and the acidic and basic amino acids account for 5% each, as shown in Table S4. Residues in TM3, TM6, and TM7 make consensus contacts with diverse ligands across class A GPCRs (at positions 3.32, 3.33, 3.36, 6.48, 6.51, and 7.39, Ballesteros–Weinstein numbering^85^).^84^ For the 31 GPCRs, most amino acids in these positions are hydrophobic, such as the conserved Trp (6.48), except for a few with polar residues such as Asn, Thr, and Ser at positions 3.33 and 7.39, besides the conserved Asp (3.32) (Table S4). Electrostatic interactions define the maximum ligand efficiency, as shown in a study investigating the properties that lead to efficient binding.^86^ Charged amino acids at the receptor surface are important in attracting or repulsing positively charged ligands in the case of aminergic GPCRs. In most cases, a negatively charged amino acid (Glu or Asp) attracts the positively charged ligand via electrostatic interaction.^87^

Previous studies failed to find highly selective M1 agonists to treat AD due to the activation of the peripheral M2 and M3 receptors, which may lead to cholinergic side effects of bradycardia, nausea, and diarrhea.^22,23^ Our models detect differences that could hopefully lead to selectivity: the M1 agonist model is dominated by 50% of partial charge descriptors. The main descriptors in the filters are the total negative/positive van der Waals (VdW) surface area and a sum of the VdW surface area in different ranges of atoms’ partial charges (Figure S16). Therefore, it differs from other muscarinic agonist models (Figures S17 and S18). The M3 agonist model has a high percentage of atom and bond count descriptors (such as the number of aromatic atoms), 5-fold larger than the percentage of that descriptor family in M1 and M2. Virtual screening results support these differences: we found 1191 molecules from Enamine DB, with index scores (>0.7) in the M1 agonist model but negative indexes in the M1 antagonist and M2/M3 agonist and antagonist models.

Another example is atypical antipsychotics, generally characterized by greater affinity to the 5-HT2A receptor, thus causing fewer extrapyramidal side effects than typical antipsychotic drugs, which have affinities to both 5-HT2A and the D2 receptors. Partial charge descriptors comprise 60% of the 5-HT2A antagonist model’s descriptors followed by 15% of pharmacophore descriptors (Figure S19). However, the D2 antagonist model is characterized mainly by adjacency and distance matrix descriptors (47%; Figure S20). Therefore, it may be possible to discover selective molecules to one of these two receptors. Virtual screening found 1138 candidates from the Enamine DB with a high index score (>0.7) at the 5-HT2A agonist model with no activity at the D2 receptor.

### Comparing Target Identification: ISE vs SEA

ISE and SEA^67−69^ are two methods that may be used to identify targets for new molecules. The classification by ISE models is based on molecular physicochemical properties. The SEA is based on comparing the topology fingerprints of a candidate molecule to the fingerprints of a set of known ligands for a target. Comparisons are performed by Tanimoto similarity, and so, if a molecule (such as a known drug) already exists in the set of ligands of a protein, then that protein will be identified as a target with high probability and with Tanimoto coefficient (Tc) = 1, full identity. The SEA is, therefore, not a classification algorithm.^68^ In general, ISE searches for needles in a haystack (of properties), while SEA looks for similar needles (by other properties).

We compared the performance of both algorithms for predicting interactions of 253 CNS drugs on 31 GPCRs. While ISE models can provide us with the activity type of molecules (agonist or antagonist), SEA can only provide data on the interaction with a specific receptor. The SEA currently covers a much wider number of targets.^67−69^ So, our current comparison between ISE and SEA is limited to GPCRs only, and future comparisons to other sets of receptors would be required to expand the current conclusions.

We find that ISE is more accurate than SEA^68^ for predicting drug-GPCR interactions for all receptors except D1, the opioid delta and kappa, and 5-HT7 receptors (Figure S15). In SEA, the average Tc for D1 is 0.7, and the success of predictions is 50%. For the opioid receptors with more than 50% success, the average max Tc ranges between 0.5 and 0.6. Meanwhile, for 5-HT7 with an average Tc of 0.5, the success rate is 31%. The similarity values mean that SEA has a greater similarity between the training sets and the CNS-screened drug.

Due to the character of searching by SEA, as mentioned above, screening DrugBank molecules by SEA artificially increases the chances for correct predictions because these drugs are compared at each target to the very same molecules. If most of a target’s ligands are known drugs, then SEA will easily identify drugs from DrugBank. This is why SEA successfully predicts the cannabinoid interactions (three interactions for each receptor, CB1, and CB2, with cannabidiol, dronabinol, and nabilone).

For the CNS GPCRs of this paper, it seems that methods based on physicochemical properties are more successful than the 2D topological daylight fingerprints used by SEA to predict interactions. The SEA covers many human and rodent targets and can build models with at least five ligands.^68^ ISE still has a limited number of models, and more than 25–30 known ligands are required to construct a model, while models with few ligands, as in the 5-HT7 agonist model, might have lesser statistical significance. Both methods suggest which molecule has a greater chance of hitting a target. Due to the need for larger sets of active candidates, if a drug molecule is included among the ISE active compounds, then it has a minor effect on the discovery of that target, even for that specific drug: the properties that distinguish active compounds from inactives are results of the inclusion of many molecules, and the contribution of a single drug is low. However, we excluded the drugs from the active molecules used to build the ISE models in all the cases discussed.

### Virtual Screening to Find Multitargeting Candidates

Ideally, one would like to design drugs that interact with specific therapeutic targets and avoid anti-targets that could lead to side effects. This requires producing more than a single-target model and screening molecular databases by more than a single model. Therefore, both target and anti-target models are essential, and any screening through more than a single model may be defined as “multitargeting”. That relatively new paradigm of multitargeting, which has gained momentum in the last decade, replaces the old “one disease, one target, one drug” with “one disease, many targets, one drug” so that a single molecule could fulfill both the pharmacodynamics and pharmacokinetic needs for biological interactions. Multitargeting gains attention in many multifactorial disease conditions such as neurological and metabolic disorders, cancer, viral, bacterial, fungal, parasite, and others.^88^ Computational methods have become a crucial component of many drug discovery programs, and VS techniques are widely used and may be easily extended to discover multitargeting candidates.

We find many potential interactions by screening molecules of the ZINC drug-like and Enamine databases, with average interactions of 6.7 and 5.5 per molecule, respectively (Figure 4A). These numbers are compatible with previous findings regarding the average drug interactions (6–7) with GPCRs.^5^ We use a much higher cutoff to pick candidates for “wet” testing, usually above 0.7. In that case, we get about 5 million molecules (from the ZINC drug-like DB) and less than half a million from the Enamine DB with predicted activity on any of the 31 models.

### Reliability, Accuracy, and Bias: Descriptors and ISE

Following a request from a reviewer, we briefly discuss the subsequent issues: the descriptors are taken from a set produced, examined, and constantly reassessed by the “Chemical Computing Group”.^82^ Numerous research groups^89−94^ have used and published those descriptors for many years.

Any error in the learning sets’ structure translates into either inability to calculate descriptors for erroneous chemical records or mistaken descriptors; this is why chemical data curation is so important.^95,96^ The data curation steps are mentioned in Data Collection and Dataset Preparation sections in the Methods section.

Examining ∼200 descriptors for thousands of molecular structures presented as SMILES “translated” from 2D structures is impossible. However, based on several structures, we check descriptors such as H-bond donors and H-bond acceptors. We also examine charge descriptors visible from the 2D structure and chemical knowledge (i.e., all amines should have neutral and charged species; all acids should have both a neutral and negative carboxylate single charge).

There is always room for improving such a complex set of activities for model building. Many issues could improve the results, starting with choosing the most reliable experimental results out of ChEMBL. We tested a few data combinations for each model and selected the top-performing models for this MS. Selecting different random sets for the same active set did not improve the models’ performance (Table 3). Selecting different descriptors (from other types of software) may enrich the subset of descriptors and affect the model’s performance. For example, we tried to construct models for CB2R agonists with other types of descriptor software (RDKit (v.202002121353),^97^ Mordred (v.1.2.0),^98^ and AlvaDesc (v.2.0.8),^99^ unpublished data) and the models built with the MOE descriptors have the best results (better MCC, EF, and AUC values).

The best way to compare ISE to other algorithms is by *in vitro* experiments on predicted candidates. We have shown in the past^100^ that ISE produced more appropriate candidates than kNN, although the statistical parameters were nearly identical.

## Conclusions

This study presents the methods and results of model construction for key 31 GPCRs involved in CNS disorders. We present agonist and antagonist activity models for each receptor, except for those without enough molecules to enable model building. We use statistics to evaluate the quality of our models and find that most of them are of very high quality and cover targets with no solved structures yet. This enables us to screen and score any molecule in the GPCR models. These scores may prioritize molecules for specific receptor activity types and disease conditions. Screening the ZINC drug-like and Enamine databases demonstrates the ability to locate multitargeting candidates. The learning sets are structurally diverse, so we expect diversity in the screening results, as proven by the predictions of novel scaffolds, confirmed by our recent discoveries of CB1R antagonists.^47^

Screening the DrugBank database yields a success of 56% in predicting activity and 68% in predicting CNS drug-GPCR interactions. ISE identifies the CNS drug target better than the SEA algorithm by relating molecular properties to preferences for interacting with GPCRs. The drug-GPCR interactions, from the DrugBank screening through our activity models, will be discussed in subsequent publications focusing on repurposing options, explaining adverse drug reactions, and suggesting new possible mechanisms of action.

## Methods

### Data Collection

#### Learning Sets

Molecular activities (agonists with EC_50_ values and antagonists with IC_50_ or *K_i_* values) at the different human GPCRs were taken from ChEMBL (http://www.ebi.ac.uk/ChEMBLdb/).^61^ The search for human targets was done by the relevant UniProt codes (Table S4). The active molecules were examined, and molecules were excluded by several criteria:

- Duplicates (having the same ChEMBL ID) were removed, keeping the molecule with the worst reported activity out of several reported with different activities.
- Molecules with comments such as “outside typical range” or “potential transcription error” were removed.
- Only explicitly defined potency values were retained, and approximate measurements such as “ >”, “<”, or “∼” were discarded.
- Molecules with activity above 100 μM were excluded. A subset of highly active molecules (i.e., activity values less than 10 or 100 nM) was retained from the larger set of reported active compounds for some models.
- Molecules with a “confidence score” (given by ChEMBL) above seven were kept.
- We excluded drugs reported in the DrugBank database (version 4.5)^66^ from the learning sets.
- Based on the calculated descriptors, we removed mutagenic (a descriptor for the presence or absence of potentially toxic groups) and reactive (a descriptor of reactive groups) molecules from the learning sets.

The active molecules for all models are provided in Data S2. Active molecules were diluted with random molecules. Our preferred ratio of “random” molecules for diluting active ones should be 1:1000 to simulate better the chances for finding active compounds in the “real world” of chemicals.^101^ However, learning sets with such large ratios require a much longer time to produce a model; therefore, we mostly compromise by using a dilution of 100:1 inactives:actives. The randoms (“decoys”) were picked from the ZINC DB,^65^ based on the applicability domain of the active compounds,^102^ including four properties for which averages ±2 STD are required for picking the randoms: molecular weight (MW), *c*log*P*, hydrogen bond acceptors (HBA), and hydrogen bond donors (HBD).

#### Screening Datasets

The DrugBank database (July 2016, version 4.5)^66^ contains 7130 drug entries. A set of 253 CNS drugs (the complete list in Table S1) was picked out of the DrugBank DB according to classes taken from Drugs.com. We used the ZINC drug-like database (17,900,742 molecules)^65^ and the Enamine HTS 2021 database (2,159,632 molecules)^73^ for VS.

### Dataset Preparation

Using “Molecular Database Wash” by MOE software (v. 2011.10),^82^ we applied “cleaning” rules to unify all the datasets’ structures.^101^ The “Wash” procedure^103^ removes minor components (such as inorganics, organometallics, and counterions), determines the protonation state, enumerates ionization and tautomer forms, and applies hydrogen adjustment.

### Descriptor Calculation

To calculate 2D descriptors (186 descriptors), we used the QuaSAR-MOE (v.2011.10).^82^ The complete descriptors list is given in Data S1.

### Removing Redundant Descriptors

Two descriptors may be highly correlated, so leaving both might be a source of confusion about the preferred mechanism. Each descriptor’s correlation with others is evaluated, and the one with a Pearson correlation coefficient >0.9 is eliminated. Descriptors with extremely low variance,^104^ i.e., if all descriptor values for all molecules equal 0 or 1, were excluded, using KNIME (v.2.10).^105^

### Drug-Protein Matrices

We downloaded the whole dataset of 7130 drugs from the DrugBank. Of those drugs, only 310 are reported with 1097 activities (DrugBank version 5.1.9, access date: January 2022). We added the following activities: (i) 115 activities for 60 drugs in ChEMBL27 (access date: November 2020),^61^ by the following keywords in their “functional assay description”: “Agonist”, “Agonistic”, “Activation”, “Antagonist”, “Antagonistic”, “Antagonism”, “Antagonists”, “Inhibitory”, and “Inhibition”. We kept activities with EC_50_, IC_50_, and *K_i_* values only below 100 μM. (ii) Two hundred twenty-seven activities for 193 drugs in the Therapeutic Target Database (TTD, access date: November 2020),^106^ we kept only human GPCRs and DrugBank molecules and picked only those with “Antagonist”, “Agonist”, and “Inhibitor” as the mode of action. Due to the overlap between the databases, the final number of activities (agonist and antagonist) is 1212 of 361 drugs.

We created a drug-target adjacency matrix to compare our results to SEA, which does not have activity annotation. Denoting the target receptor set as T = {t1, t2, ..., tm} and the drug set as D = {d1, d2, ..., dn}, the drug-target (DT) binary interactions can be described as a bipartite DT graph G(D, T, E), where E = {eij: di ∈ D, tj ∈ T}. A link is drawn between di and tj only if the drug di interacts with the target tj (either reported or received a positive score in a particular model). The DT bipartite network can be presented by an n × m adjacency matrix {aij}, where aij = 1 when di and tj interact; otherwise, aij = 0. In total, 1165 drug-GPCR interactions were found between 361 drugs (out of the 7130 DrugBank DB) and the 31 GPCRs. For the list of 253 CNS drugs, 529 interactions were found between 112 drugs and the 31 GPCRs.

Compared to DrugBank-reported interactions, a molecule-target matrix was produced for both ZINC and Enamine database screening results. A molecule-target interaction gets a “1” whether agonist or antagonist, if the molecule received an index above zero in the particular model, while the lack of predicted interaction gets a “0”.

### Measuring Classification Performance

The Matthews correlation coefficient (MCC)^64^ (eq 1) is ideal for classification problems where the number of items in each class may differ substantially. In processing descriptors and filters, we seek to maximize true positives (TP) and reduce the false positives (FP).The MCC is a correlation coefficient between the observed and predicted binary classifications. True Positives, TP; True Negatives, TN; False Negatives, FN; False Positives, FP
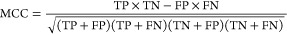
1

The receiver operating characteristic (ROC) curve is used for overall classification performance compared to other classification methods.^107^ The curve is plotted by taking the true positive rate (TPR, a probability of detection) on the *y* axis and the false positive rate (FPR, a probability of false detection) on the *x* axis, and the characteristics of the curve provide easier recognition of the precision of prediction (eqs 2 and 3).^107^True Positive Rate (Sensitivity)
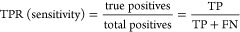
2False Positive Rate
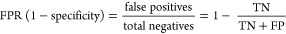
3

The ideal classifier will display a maximal area under the curve (AUC) in which positives are correctly predicted, and false predictions are minimal. It is crucial in VS for “early detection” of the positives at the beginning of the screening. We consider a “good” AUC if the numbers are above 0.85.

Enrichment factor (EF): the EF is a measure of the ability of a classification model—at specified values of indexes—to identify TP compared to random picking so that the remaining portion of the database is richer in “hits” than the initial database (eq 4).^108^Enrichment Factor Equation, where *N*_Positives_ Is the Total Number of Active Compounds in the Database and *N*_Total_ Is All the Set Compounds
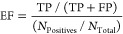
4

### Generating Iterative Stochastic Elimination (ISE) Models

**Figure 5:**
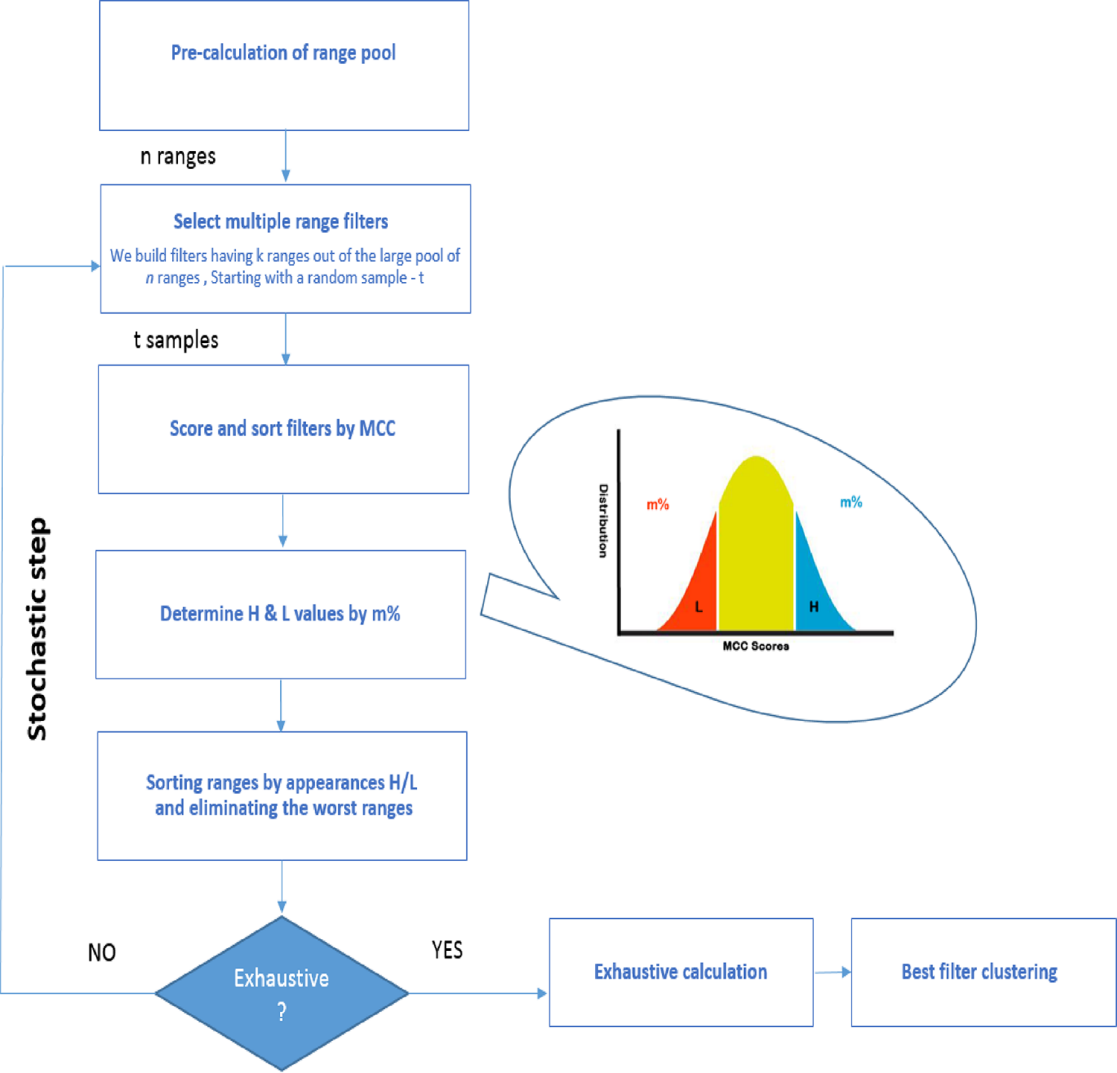
ISE algorithm. The search begins by randomly picking one value for each of the descriptors. The MCC scores all ranges of each descriptor to determine which range is best for distinguishing between active and inactive molecules. Following this step, we remain with one range of each descriptor, i.e., *n* ranges. The stochastic search is iteratively performed until we reach below 10^6^ combinations, allowing an exhaustive search of all remaining combinations. Starting with a random sample *t* (tests) of filters. The iteration is achieved by repeating the random picking and evaluation (scoring) if each variable’s value has been picked enough times to decide its contribution in all parts of the spectrum of scores. Consistently, values with bad scores on the regions of low and high values in the virtual histogram of scores will be eliminated. Subsequent iterations continue, and the number of combinations decreases until reaching the point from which an exhaustive search for all combinations can be performed in reasonable computer time. This final step allows sorting all the combinations to achieve a final set of ranked solutions-filters.

ISE has been described in detail previously.^49,56^ Currently, ISE is mainly employed to construct classification models of molecular bioactivity as a basis for VS to find novel and diverse bioactive candidates.^50,79^ This type of problem is characterized by a highly complex combinatorial nature due to having many variables (descriptors), each with many values that form numerous ranges of descriptor values (Figure 5).

The models generated by ISE are a large set of “filters”—each filter consists of five ranges of physicochemical descriptors. A model may contain hundreds and more filters, each having a different ability to distinguish between active compounds and inactives as measured by its MCC value. We limited our models to the top 1000 MCC-scored filters or the top 20% MCC values.

#### Five-Fold Cross-Validation

The “learning set” is divided into four training and one test set by randomly constructing five groups, each with the same proportion of active compounds and inactives.^62^ Modeling is performed with four out of the five groups, while the test set is used for validation. Once all groups (“folds”) were used and scored as test sets, we have all molecules’ scores in the learning set. In cases where the total number of active compounds is small, i.e., below 20 (the 5-HT7 and H1 agonist models), we employed the leave-one-out (LOO) cross-validation.^63^ The validation process consists of comparing the folds’ MCC, AUC, and other characteristic values.

#### Ranking Molecules

Screening any set of molecules through the filters allows ranking them by the addition or subtraction of scoring by each filter—a molecule that passes a filter gains its score, while it loses that score if it does not pass. Normalization by the number of filters limits the final score of each molecule to be between −1 and + 1 (eq 5):^49,56^If the molecule complies with all filter ranges, then it gets a positive weight (δ_positive_ = 1, δ_negative_ = 0). If it does not comply with one or more ranges of that filter, then it gets a negative weight (δ_positive_ = 0, δ_negative_ = 1). The score is the sum of the weights of all the filters divided by the number of filters (*n*). The *F*-score refers to the harmonic mean of recall and precision, where recall refers to the accuracy of real prediction, and precision defines the accuracy of a predicted class
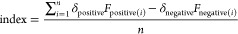
5

The higher the index, the greater the probability of a molecule being discovered experimentally as active. We choose an index cutoff for VS based on EF and TP/FP values. Huge libraries are filtered quickly, allowing us to pick a small-enriched set for further testing.

### 2D Similarity Measures

The Tanimoto coefficient (Tc,eq 6) is based on comparing “fingerprints” between two molecules. In KNIME (v.2.10),^105^ we generated the fingerprints using RDKit (an open-source cheminformatics toolkit written in C++),^97^ atom-pair fingerprints, and the CDK toolkit that generated the fingerprint similarity matrix.Tanimoto Coefficient: *N*_a_ is the number of features (bits) in the fingerprint of molecule A, *N*_b_ is the number of features in molecule B, and *N*_c_ is the number of features common to A and B. Each feature is indicated by its presence (1) or absence (0) within a molecule 0 ≤ *T* ≤ 1
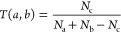
6

### Sequence Alignment of the Binding Site Residues

Human sequences of 149 binding site residues for the 31 GPCRs were downloaded from the GPCRdb (https://gpcrdb.org/).^3^ The residues were divided into five types: nonpolar and aliphatic (GLY, ALA, VAL, LEU, ILE, MET, and PRO), aromatic (PHE, TYR, and TRP), polar and uncharged (SER, THR, CYS, ASN, and GLN), acidic (GLU and ASP), and basic (ARG, HIS, and LYS) amino acids.

### Similarity Ensemble Approach (SEA)

The SEA identifies targets by comparing a candidate to a set of known ligands for that target and presents their order of probability (*P*-value) to suggest the likelihood of hitting that target.^67^ Different types of molecular fingerprints may construct SEA models. To screen the 253 CNS drugs, we used fingerprints from their library (rdkit_ecfp) with an affinity threshold of 5 nM (default settings). To compare the results with reported interactions, we kept only interactions with human GPCRs and *P*-values less than 10^–5^.

## Data Availability

All relevant data are part of the Supporting Information. The ISE algorithm was published in J. Israel Chemistry special issue for the Nobel Prize in Computational Chemistry in 2013. Stern and Goldblum (10.1002/ijch.201400072 Appendix A). The algorithm is briefly described in the Methods section. The active molecules used to build the learning sets are provided.

## Supporting Information Available

Figures S1–S12: Descriptors’ distribution for GPCR families. Figure S13: The success of activity predictions of the DrugBank molecules compared to the original reported and randomized matrices. Figure S14: Heatmap for the screening results of the 253 CNS drugs through the 59 activity models. Figure S15: The percentage of successful predictions by ISE (above an index of 0) and SEA (*P*-value < 10^–5^) for the 31 GPCRs. Figures S16–S20: Detailed 2D descriptors (%) for the muscarinic (M1-M3) agonist and 5-HT2A/D2 antagonist models (PDF)
Table S1: The 253 CNS drugs used for screening. Table S2: Reported activities for drugs, as reported in DrugBank (5.1.9), ChEMBL27, and Therapeutic Target Database (TTD, access date: November 2020). Table S3: The performance values of 59 GPCR activity models. Table S4: Multiple sequence alignment of the ligand-binding sequences of class A for the 31 GPCRs (XLSX)
Data S1: the complete list of the 2D descriptors calculated by MOE 2011 (PDF)
Data S2: the active molecules used to build the training sets for each model (ZIP)

## Abbreviations

GPCRs: G protein-coupled receptors
CNS: central nervous system
7TM: seven-transmembrane
Ach: acetylcholine
5-HTR: 5-hydroxytryptamine serotonin receptors
5-HT2B: 5-hydroxytryptamine 2B
VS: virtual screening
ISE: iterative stochastic elimination
CB1: cannabinoid 1
AD: Alzheimer’s disease
ADR: adverse drug reactions
DB: database
APD: applicability domain
MW: molecular weight
HBA: hydrogen bond acceptors
HBD: hydrogen bond donors
MCC: Matthews correlation coefficient
ROC: the receiver operating characteristic
TPR: true positive rate
FPR: false positive rate
AUC: area under the curve
EF: enrichment factor
LOO: leave-one-out
Tc: Tanimoto coefficient
SEA: the similarity ensemble approach
VdW: van der Waals
RF: random forest
XGBoost: extreme gradient boosting
SVMs: support vector machines
MLP: multilayer perceptron
DNN: deep neural networks
