# Supplementary material for: Activity Models of Key GPCR Families in the Central Nervous System: A Tool for Many Purposes
Source: J Chem Inf Model. 2023 May 31;63(11):3248–62. doi: 10.1021/acs.jcim.2c01531 (PMC10268961; doi:10.1021/acs.jcim.2c01531)
Supplement: Supplementary file 1 — ci2c01531_si_001.pdf [file ci2c01531_si_001.pdf]

Supporting Information

Activity models of key GPCR families in the CNS: A tool for many purposes

Shayma El-Atawneh*# and Amiram Goldblum*#

*Molecular Modelling and Drug Design Lab, Institute for Drug Research and Fraunhofer Project Center for Drug Discovery and Delivery, Faculty of Medicine, The Hebrew University of Jerusalem, Israel

#Address correspondence to: Shayma El-Atawneh, shayma.el-atawneh@mail.huji.ac.il; Amiram Goldblum, amiramg@ekmd.huji.ac.il

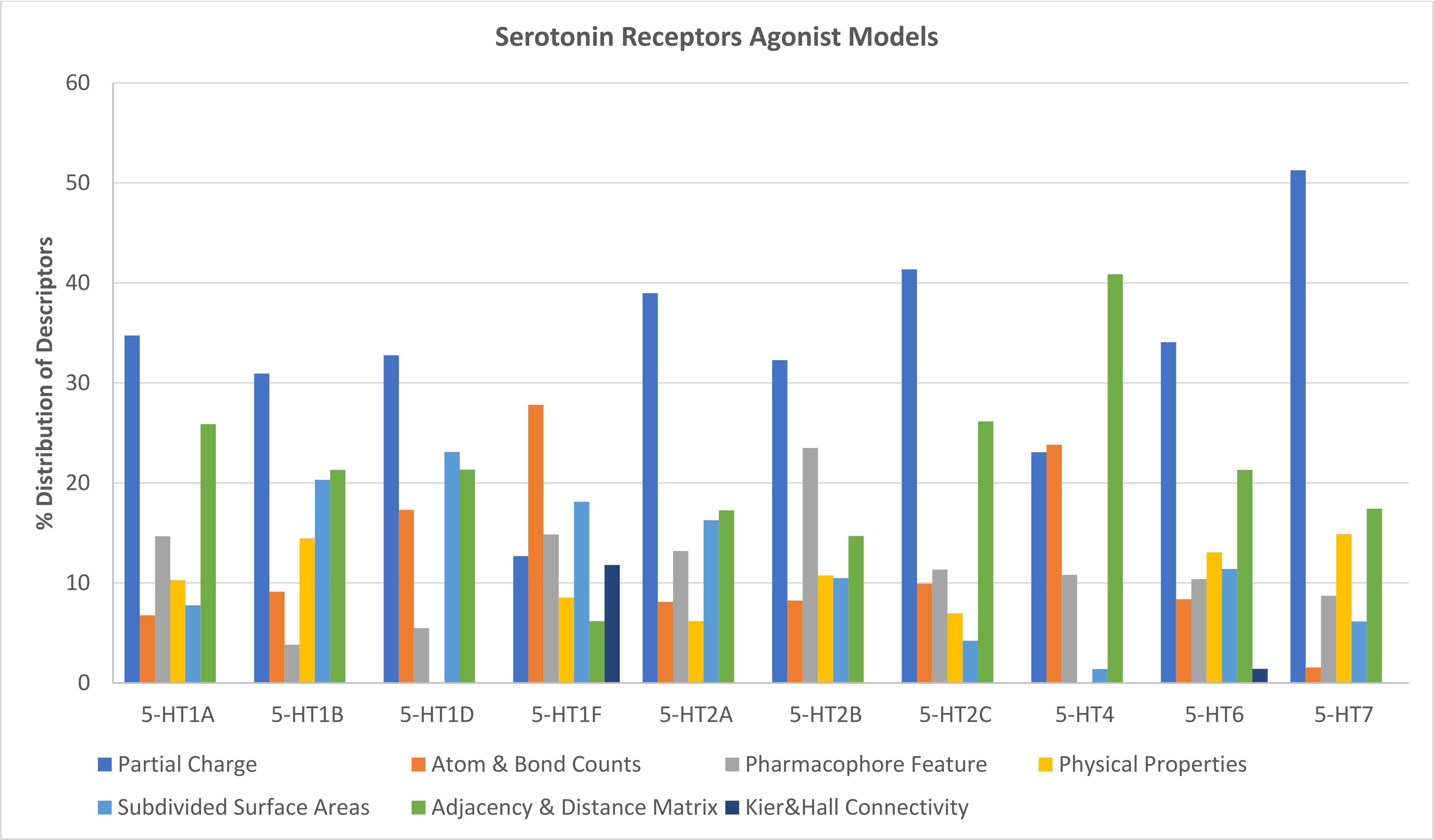

Figure S1 - Descriptors' distribution (%) for the Serotonin agonist models

The distribution (%) of the 2D descriptor-families (MOE v.2011.10)

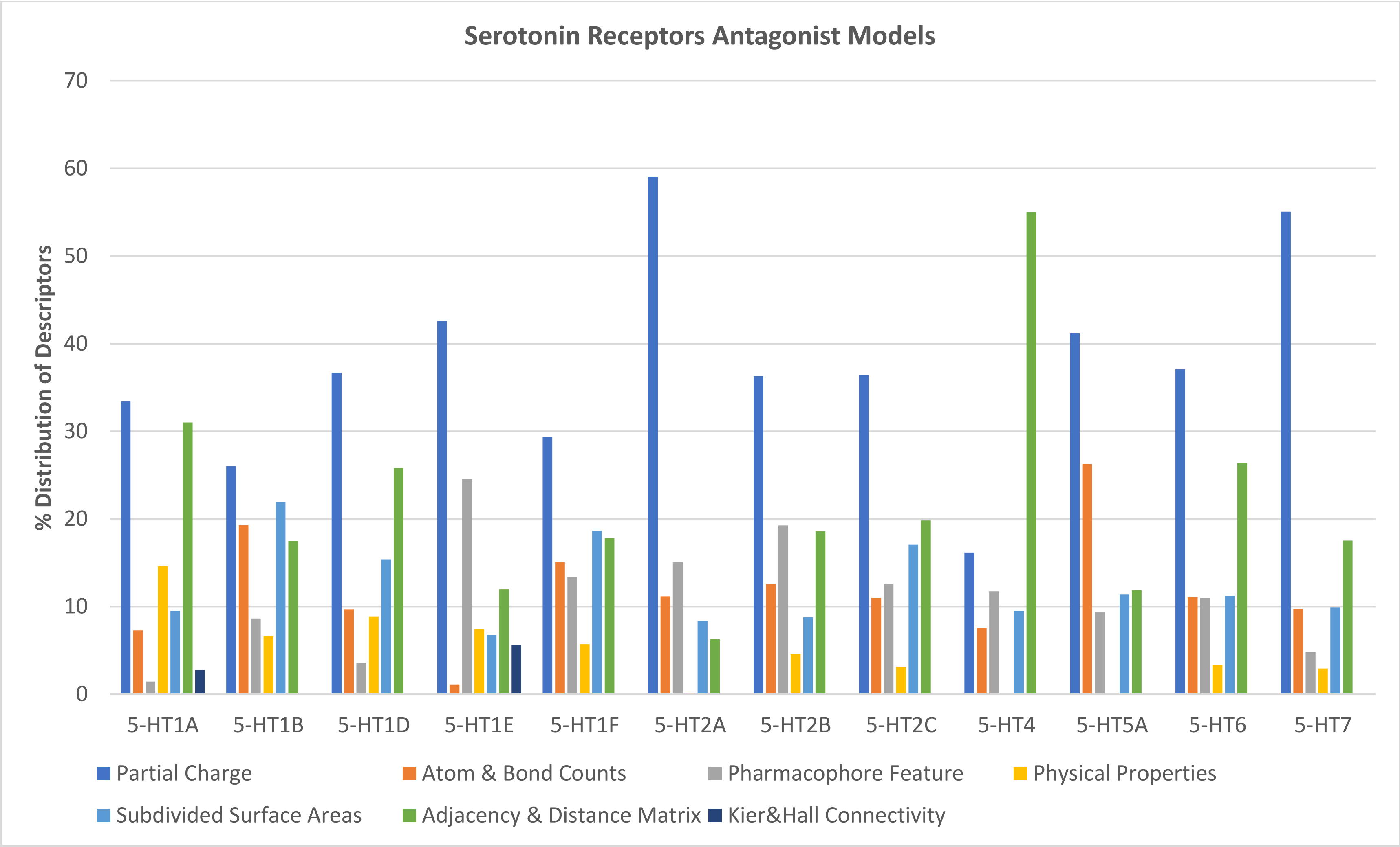

Figure S2 - Descriptors' distribution (%) for the Serotonin antagonist models

The distribution (%) of the 2D descriptor-families (MOE v.2011.10)

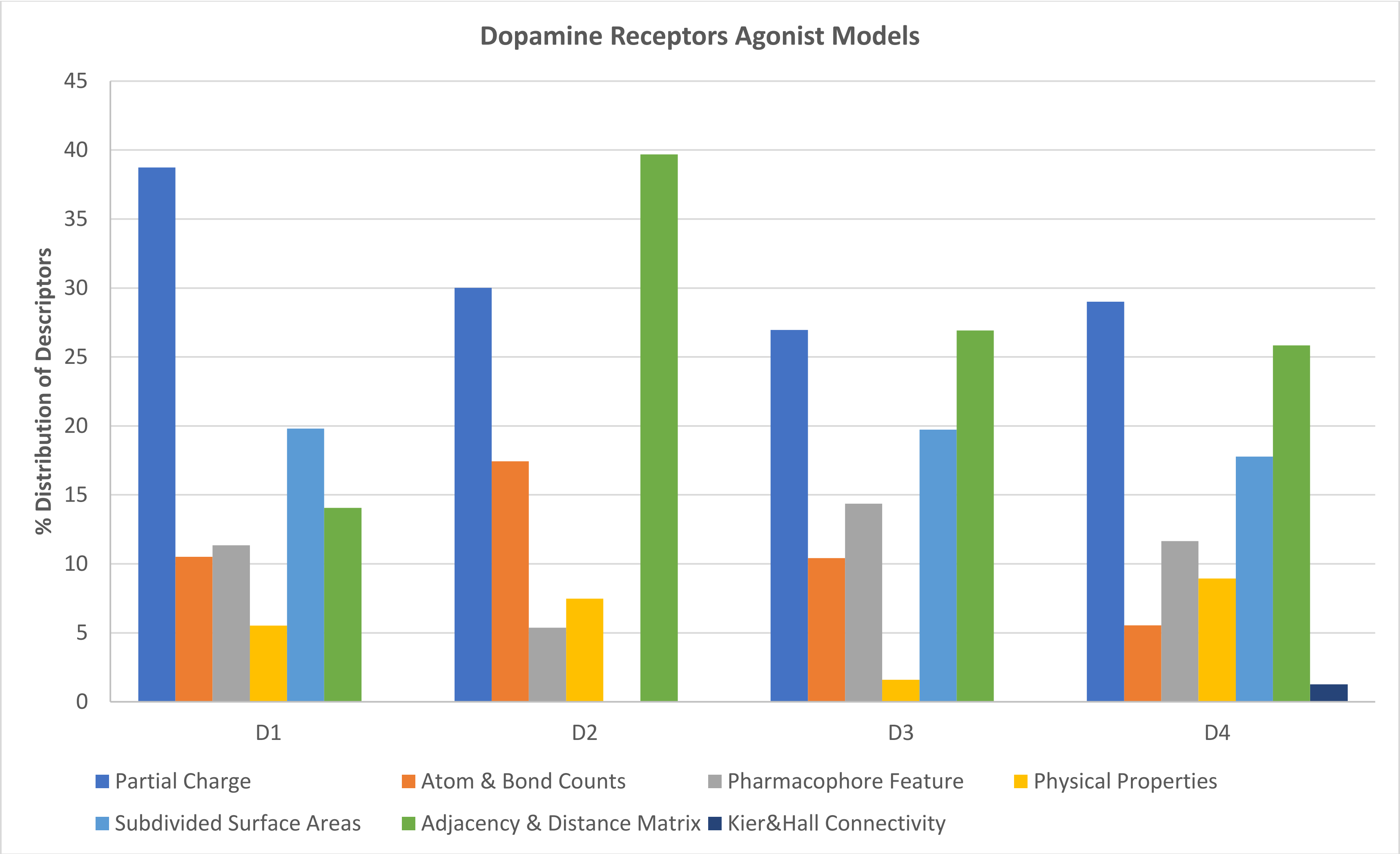

Figure S3 - Descriptors' distribution (%) for the Dopamine agonist models

The distribution (%) of the 2D descriptor-families (MOE v.2011.10)

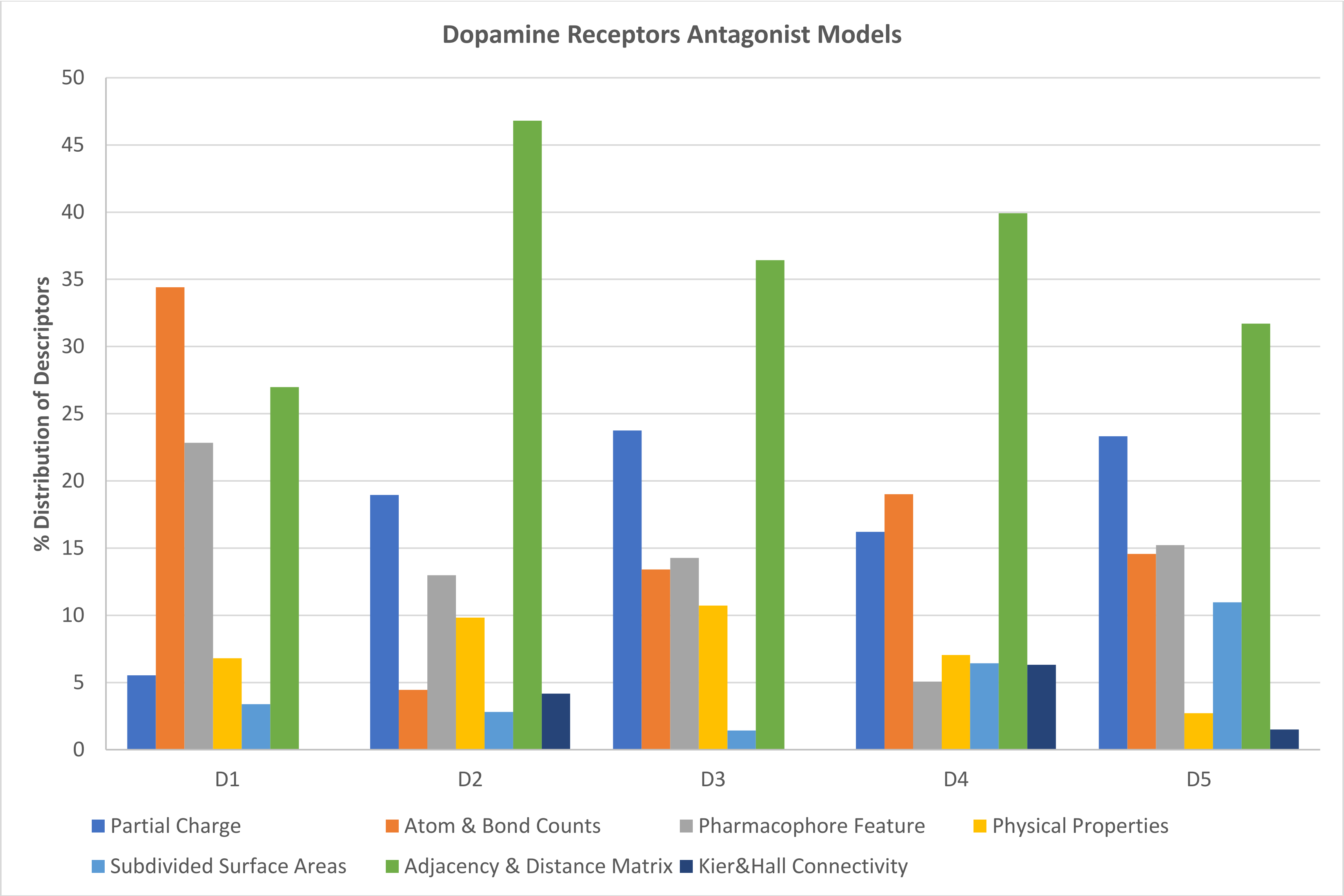

Figure S4 - Descriptors' distribution (%) for the Dopamine antagonist models

The distribution (%) of the 2D descriptor-families (MOE v.2011.10)

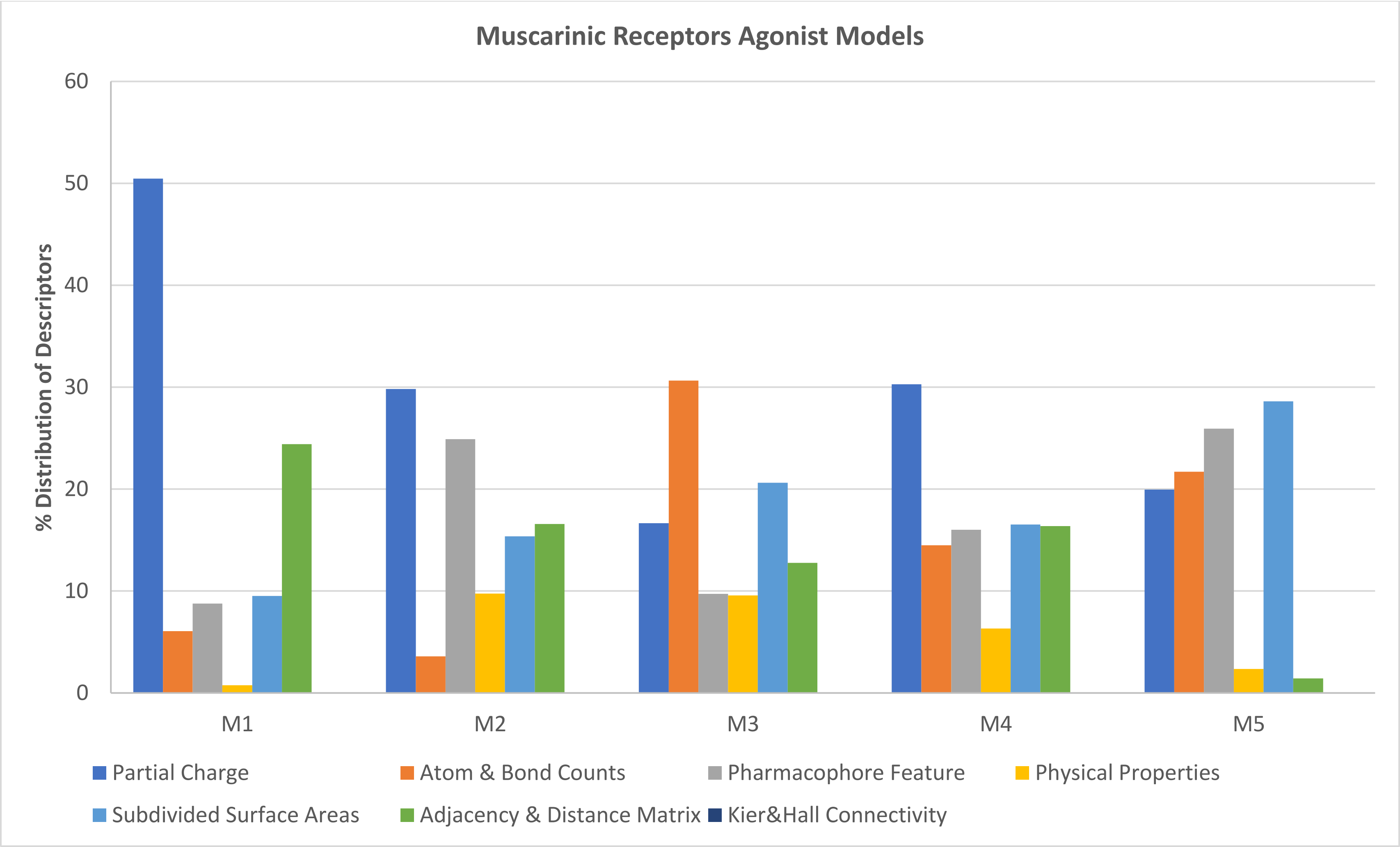

Figure S5 - Descriptors' distribution (%) for the Muscarinic agonist models

The distribution (%) of the 2D descriptor-families (MOE v.2011.10)

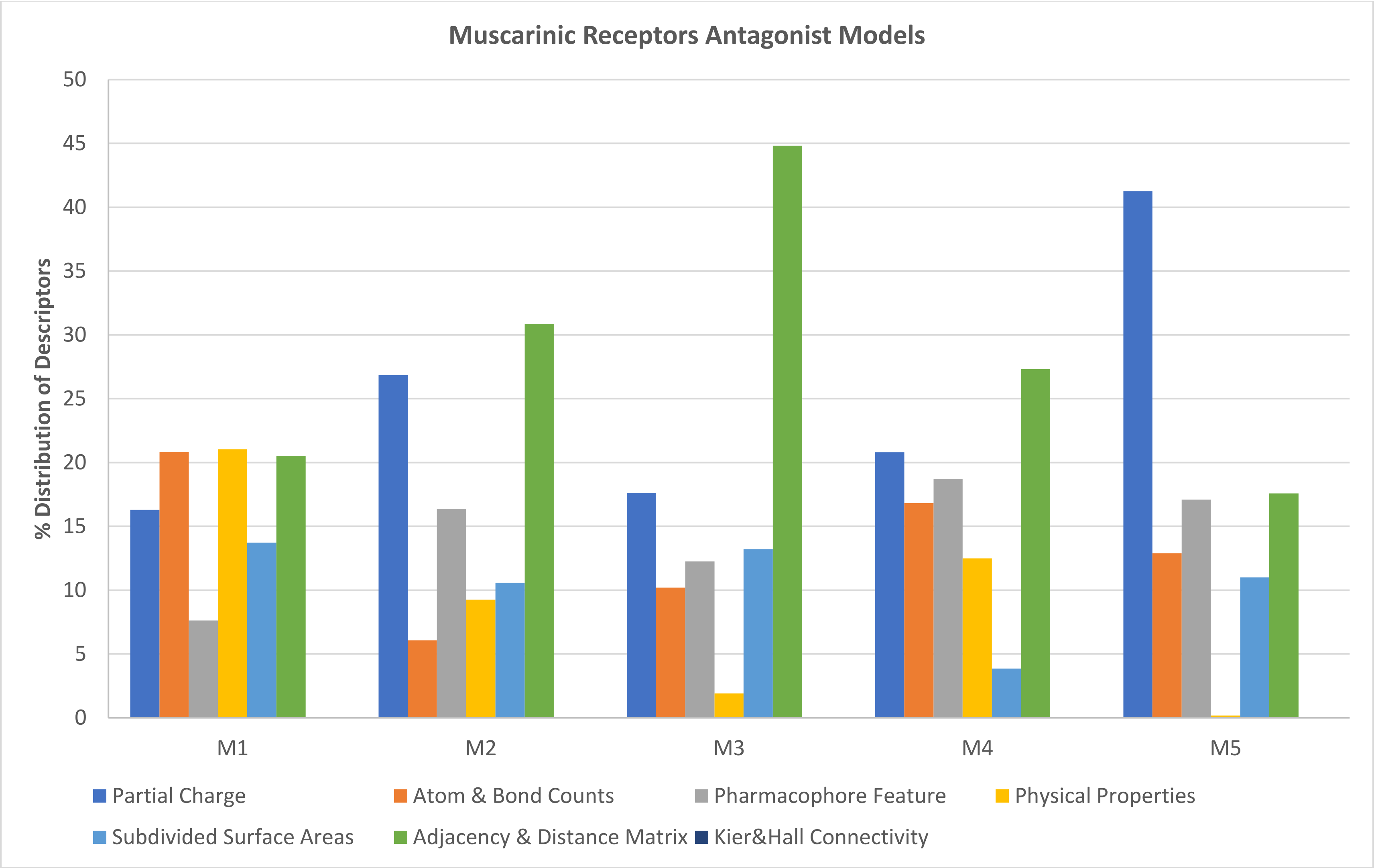

Figure S6 - Descriptors' distribution (%) for the Muscarinic antagonist models

The distribution (%) of the 2D descriptor-families (MOE v.2011.10)

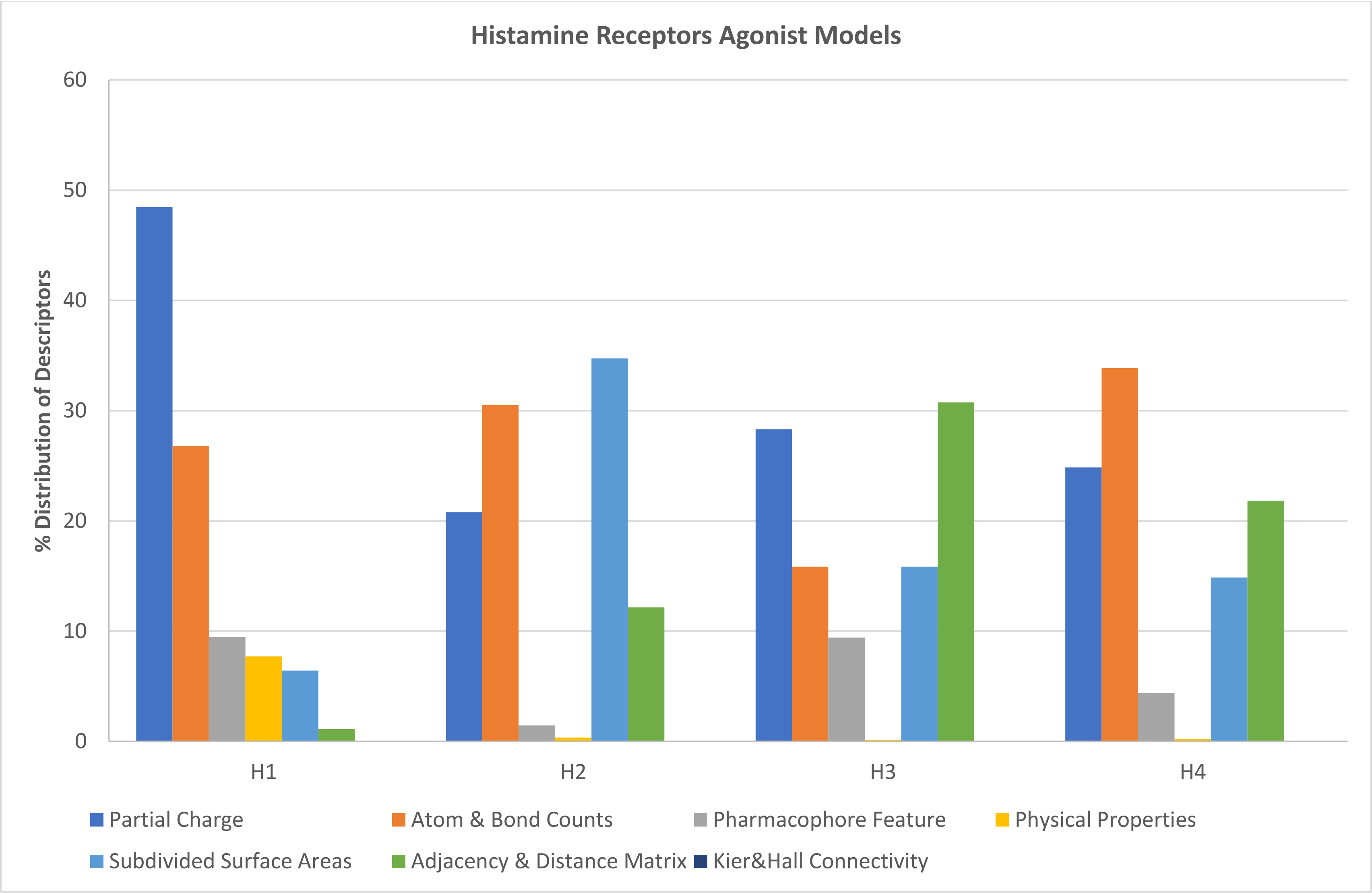

Figure S7 - Descriptors' distribution (%) for the Histamine agonist models
The distribution (%) of the 2D descriptor-families (MOE v.2011.10)

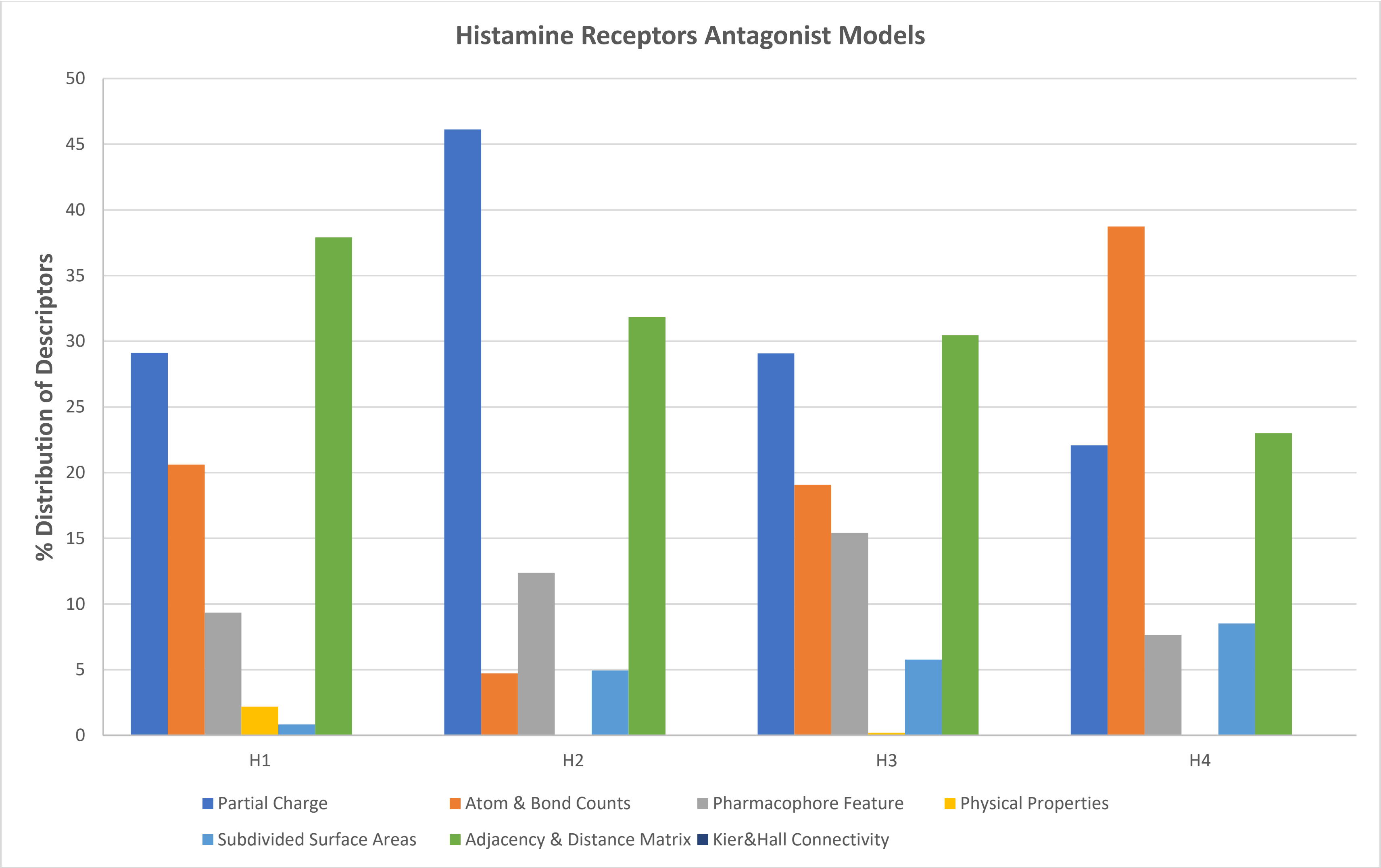

Figure S8- Descriptors' distribution (%) for the Histamine antagonist models

The distribution (%) of the 2D descriptor-families (MOE v.2011.10)

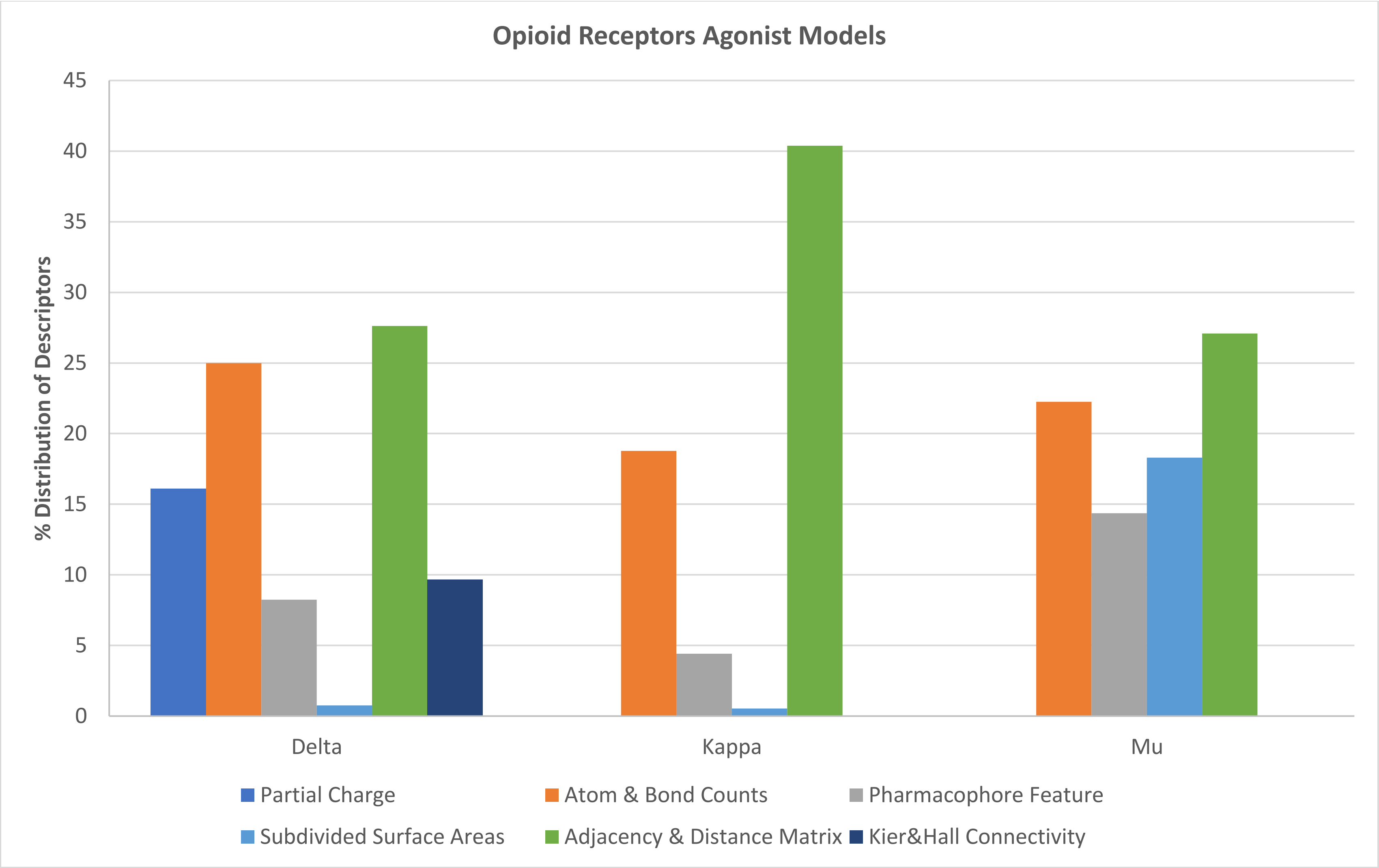

Figure S9 - Descriptors' distribution (%) for the Opioid agonist models

The distribution (%) of the 2D descriptor-families (MOE v.2011.10)

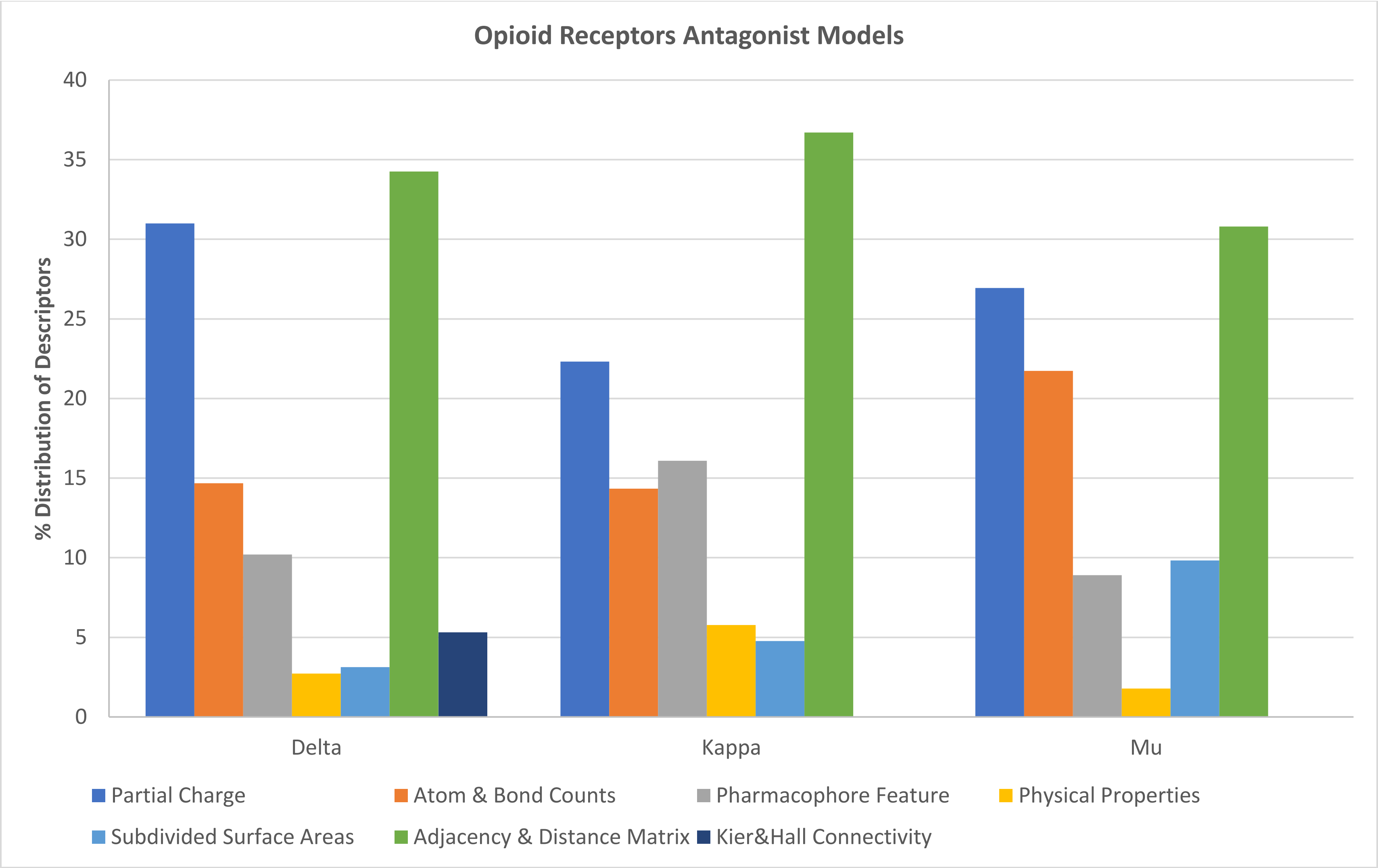

Figure S10 - Descriptors' distribution (%) for the Opioid antagonist models

The distribution (%) of the 2D descriptor-families (MOE v.2011.10)

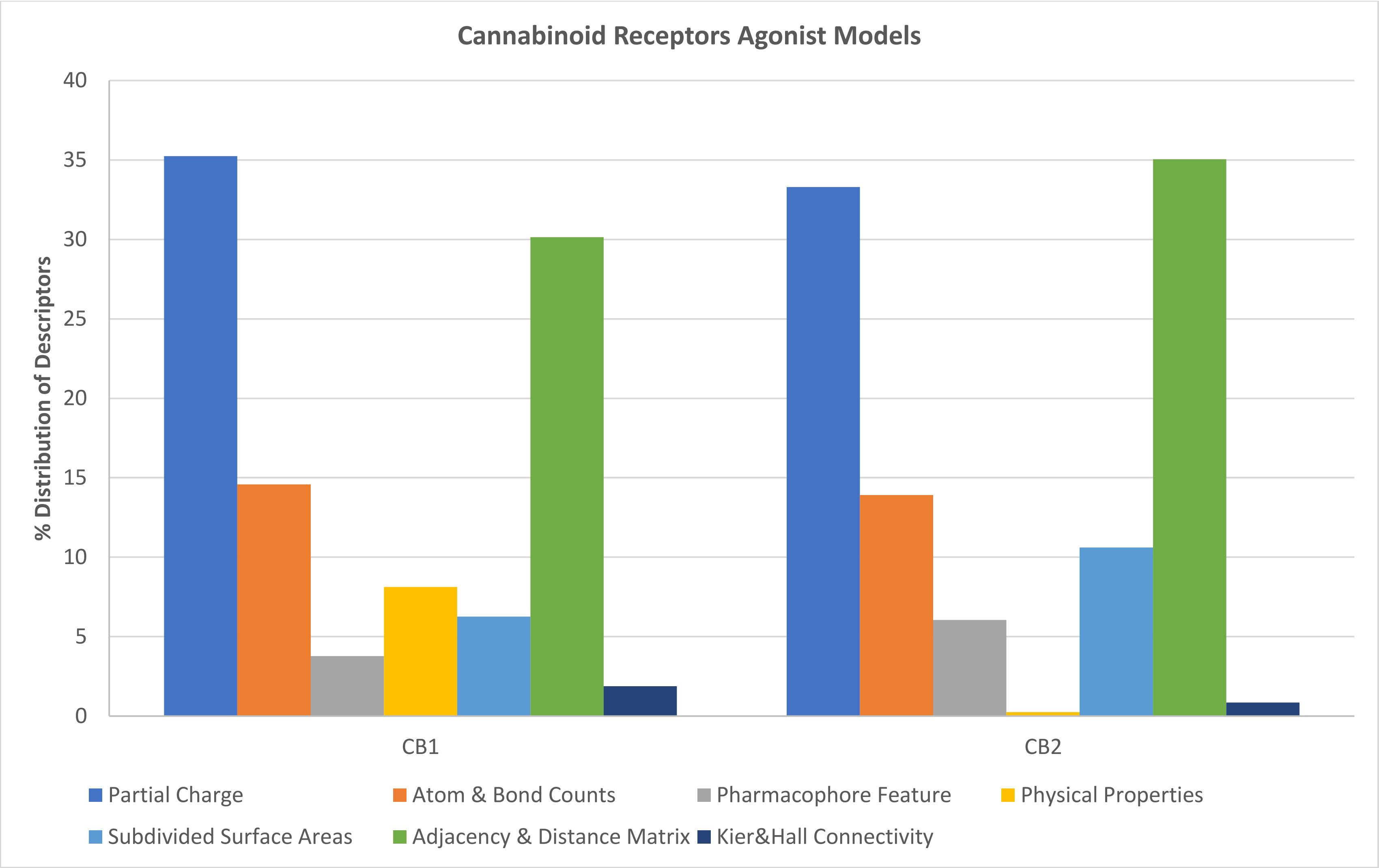

Figure S11 - Descriptors' distribution (%) for the Cannabinoid agonist models
The distribution (%) of the 2D descriptor-families (MOE v.2011.10)

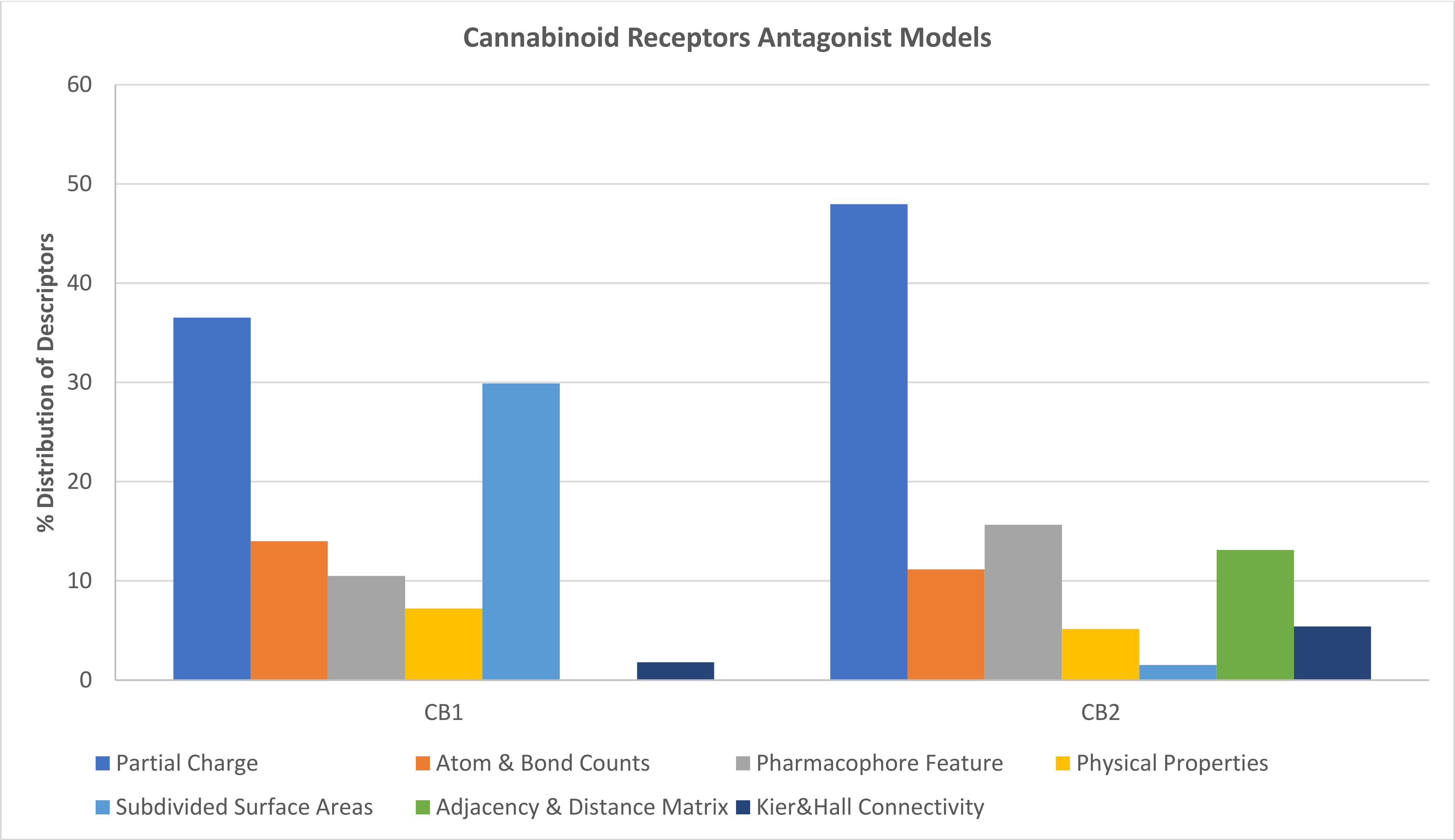

Figure S12 - Descriptors' distribution (%) for the Cannabinoid agonist models

The distribution (%) of the 2D descriptor-families (MOE v.2011.10)

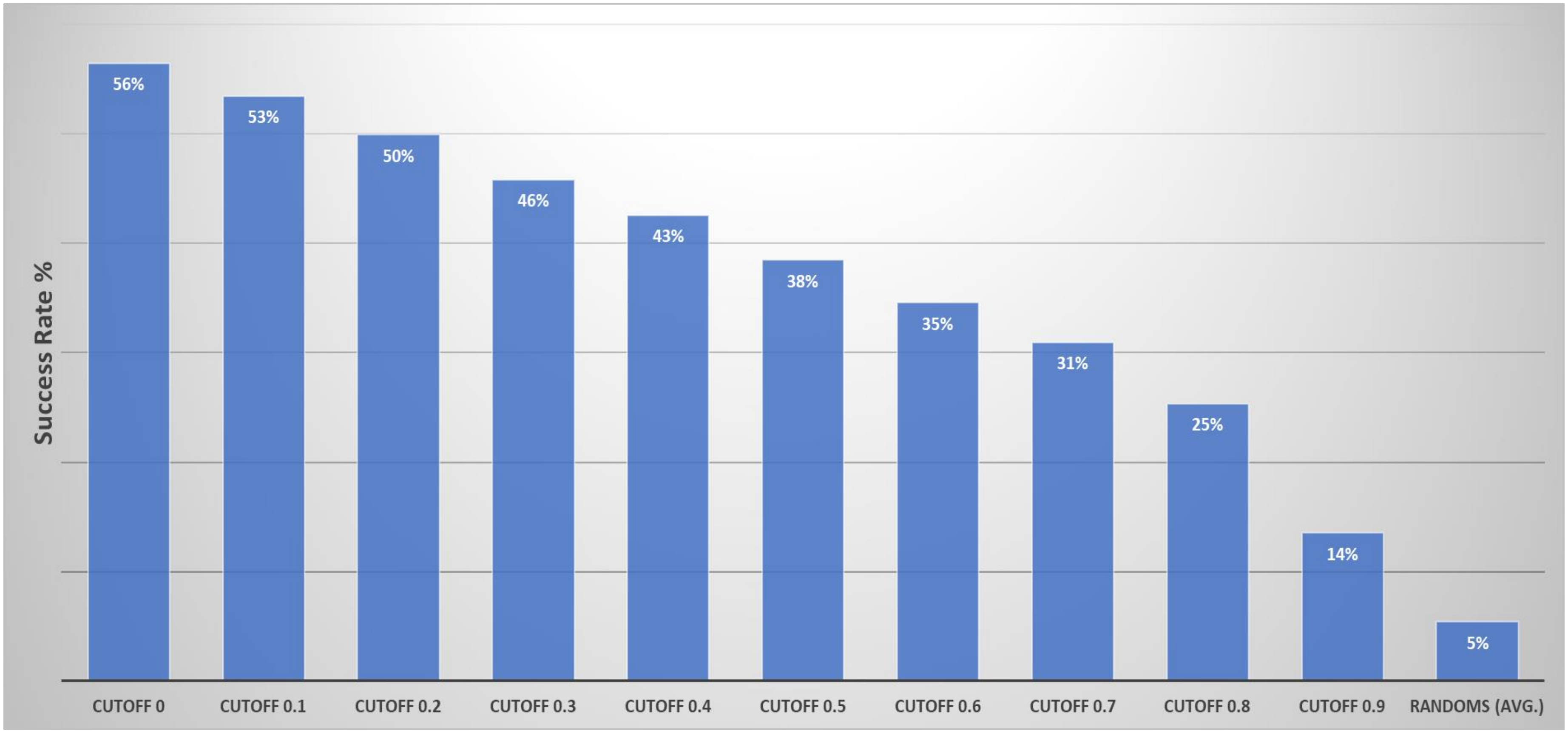

Figure S13 - The success of activity predictions of the DrugBank molecules compared to the original reported and randomized matrices

The success rate at different ISE positive index cutoffs compared to the reported 1212 activities (361 drugs from DrugBank), and the average success rate of the models compared to 1000 Y-randomized matrices.

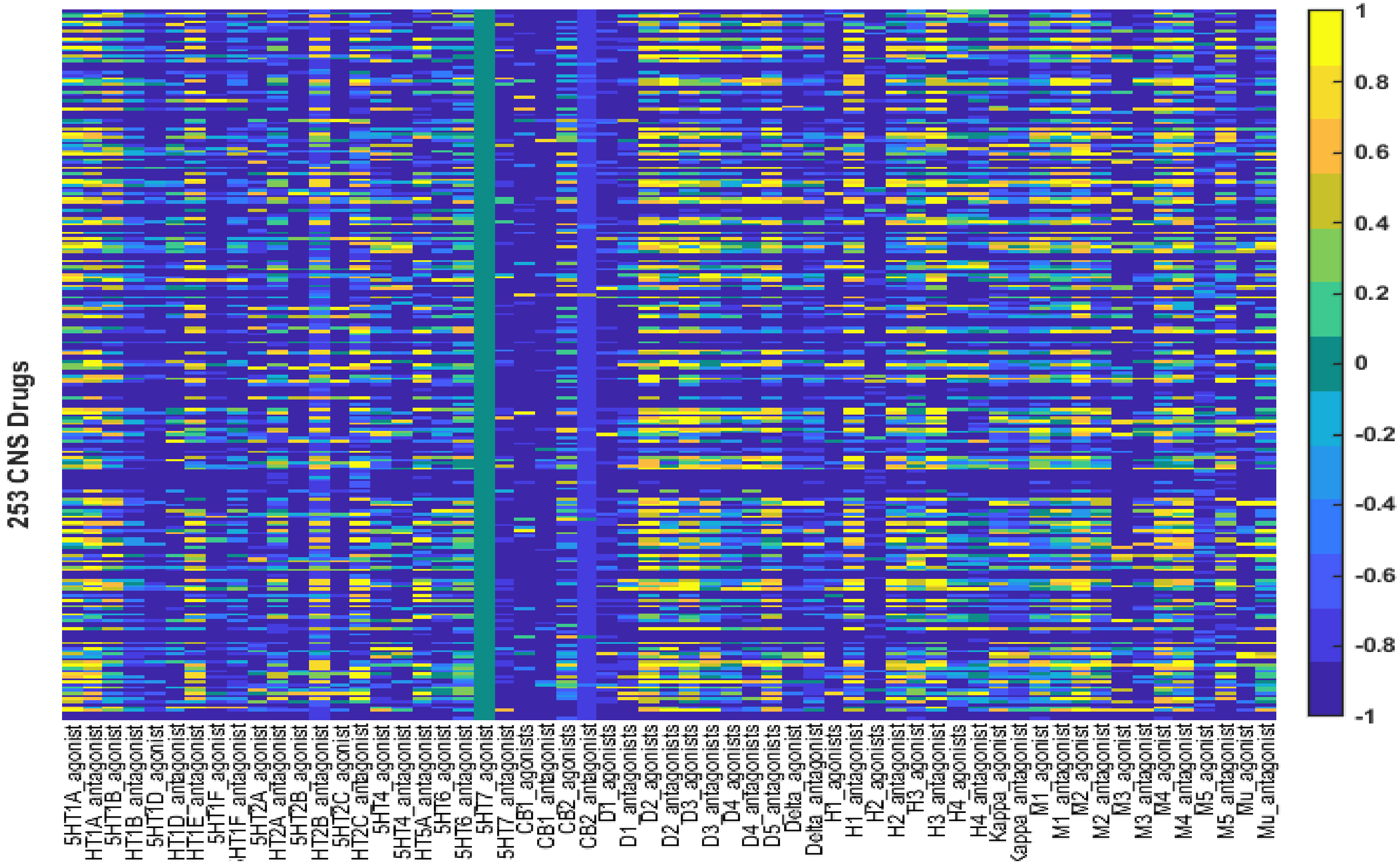

Figure S14 - Heat map for the screening results of the 253 CNS drugs through the 59 activity models

The index scores range from -1 (dark blue) to +1 (yellow). The higher the index score is, the more likely this drug is to have an activity on a specific GPCR.

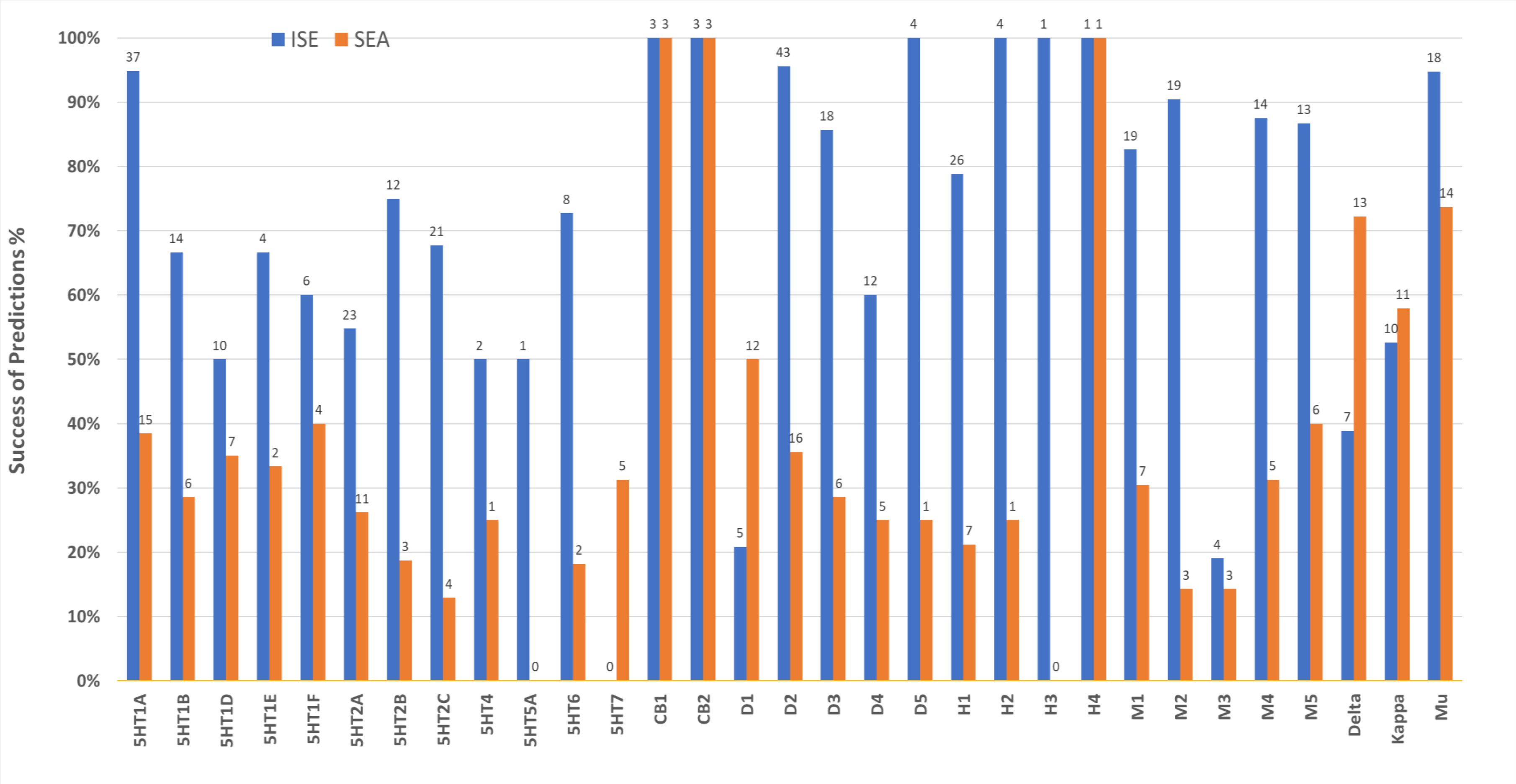

Figure S15 - The percentage of successful predictions by ISE (above an index of 0) and SEA (P-value < E -5) for the 31 GPCRs

Screening the 253 CNS drugs (washed SMILES) through ISE models and SEA server. The TP predictions are shown above the bars for each method.

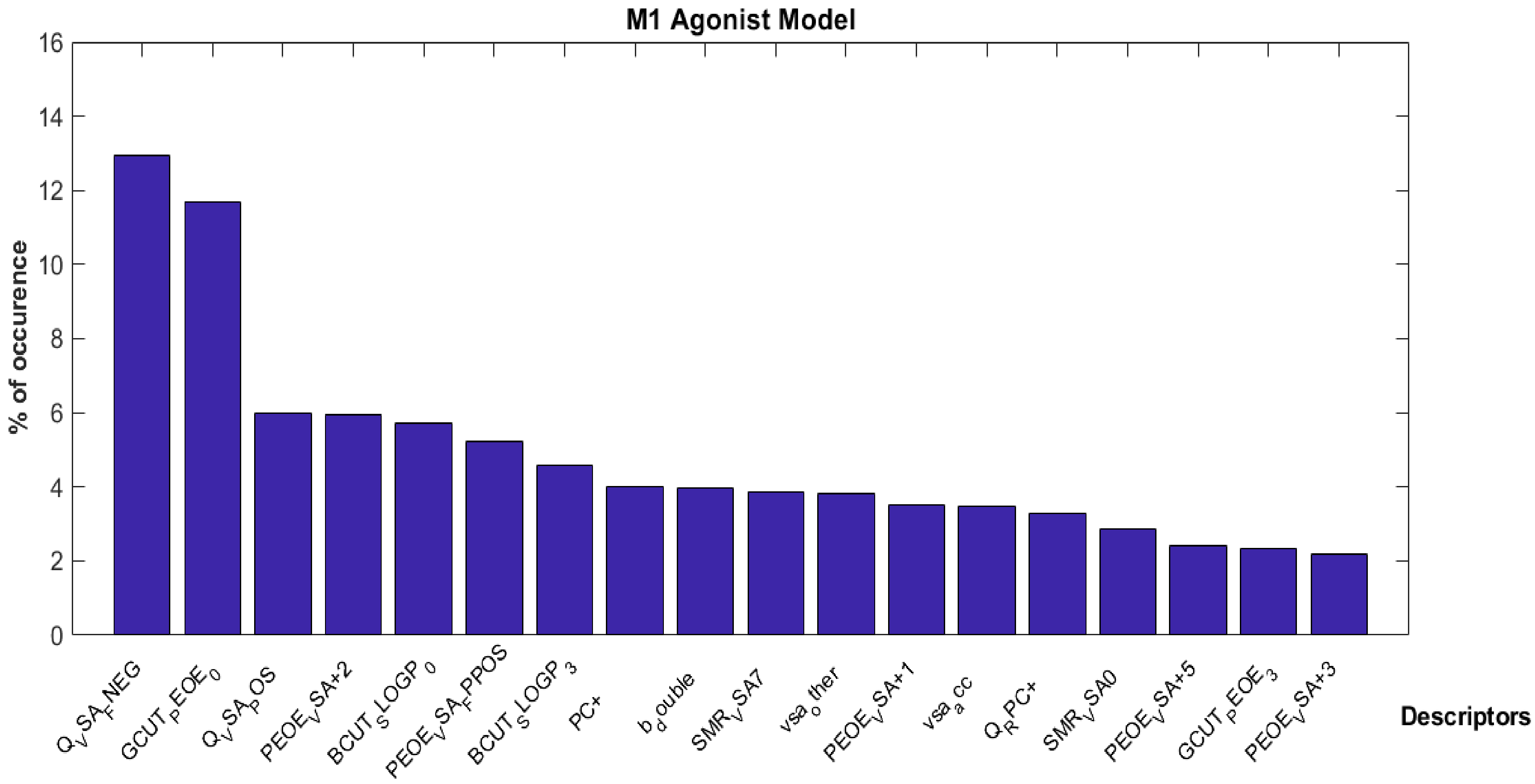

Figure S16 - Detailed 2D descriptors (%) for Muscarinic M1 agonist model

2D descriptors (MOE v.2011.10) with occurrence above 2%

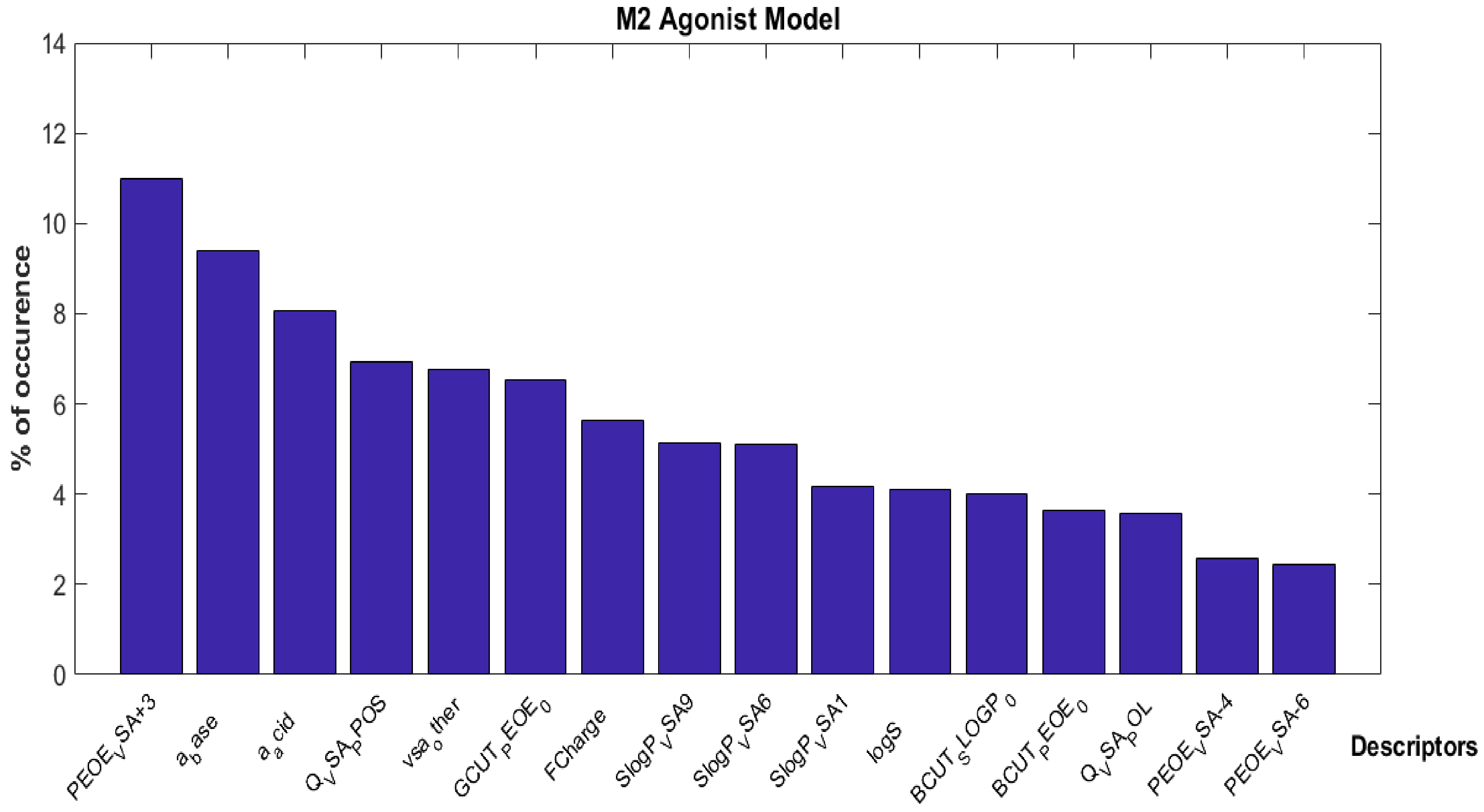

Figure S17 - Detailed 2D descriptors (%) for Muscarinic M2 agonist model

2D descriptors (MOE v.2011.10) with occurrence above 2%

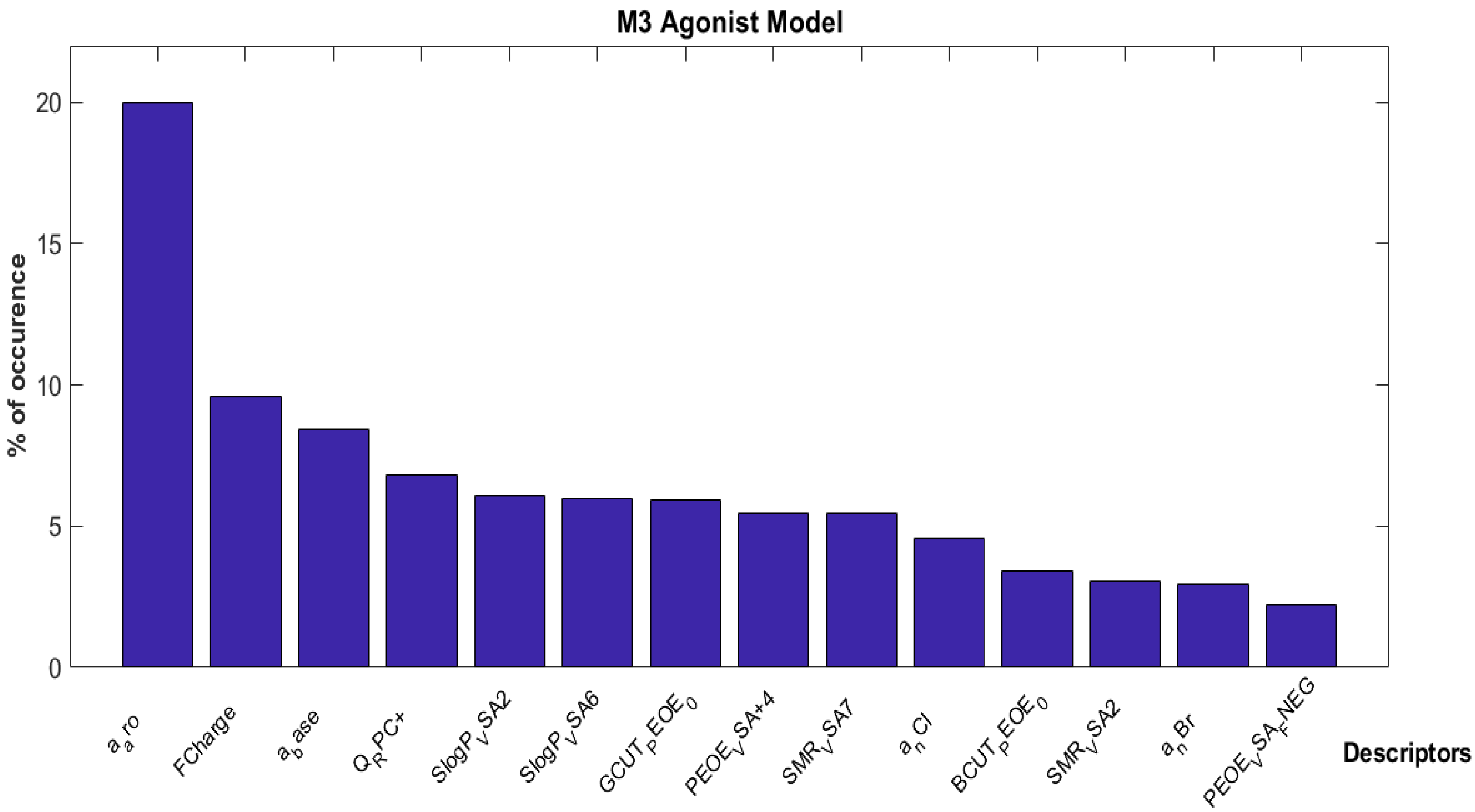

Figure S18 - Detailed 2D descriptors (%) for M3 agonist model

2D descriptors (MOE v.2011.10) with occurrence above 2%

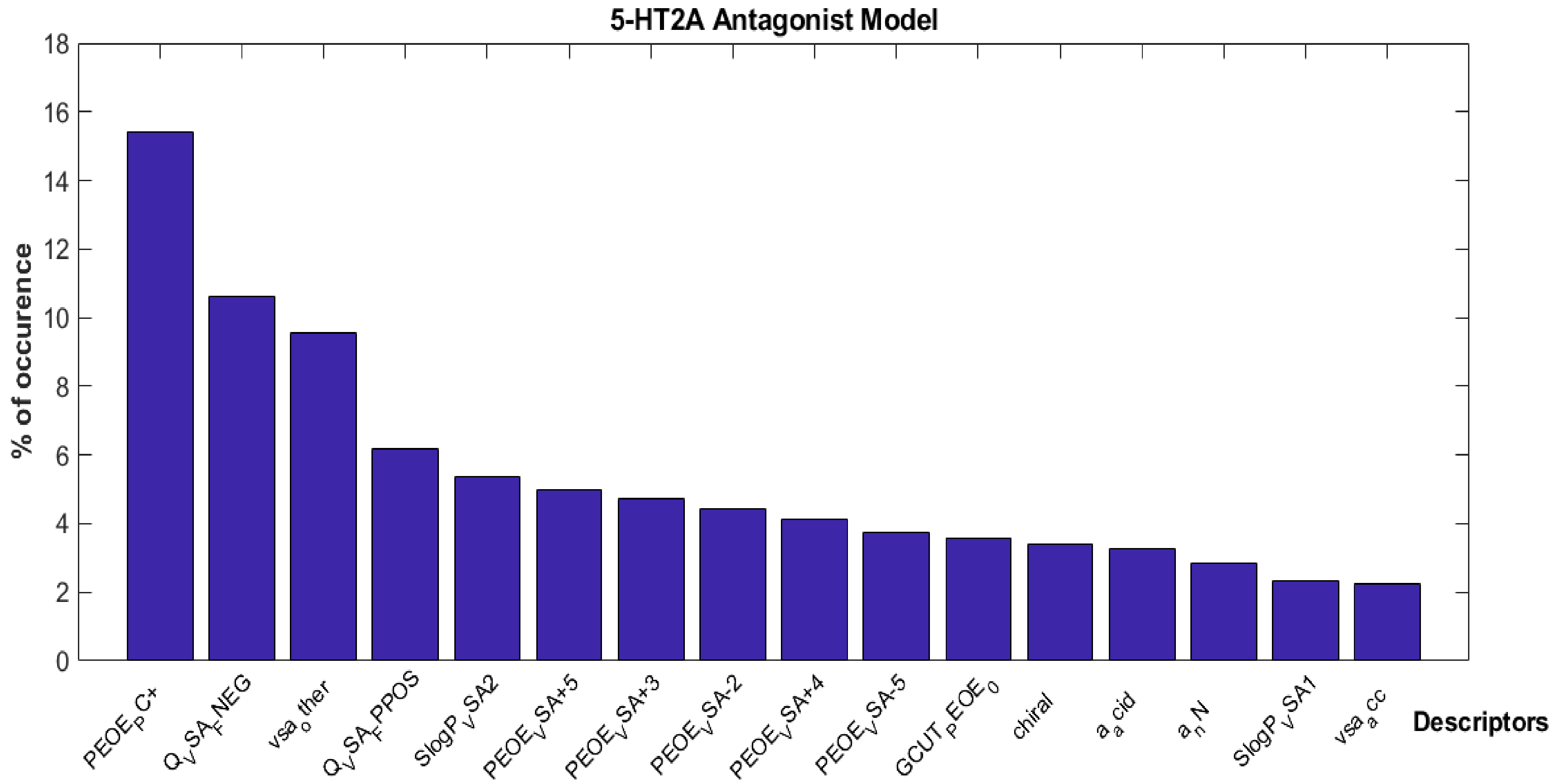

Figure S19 - Detailed 2D descriptors (%) for 5-HT2A antagonist model

2D descriptors (MOE v.2011.10) with occurrence above 2%

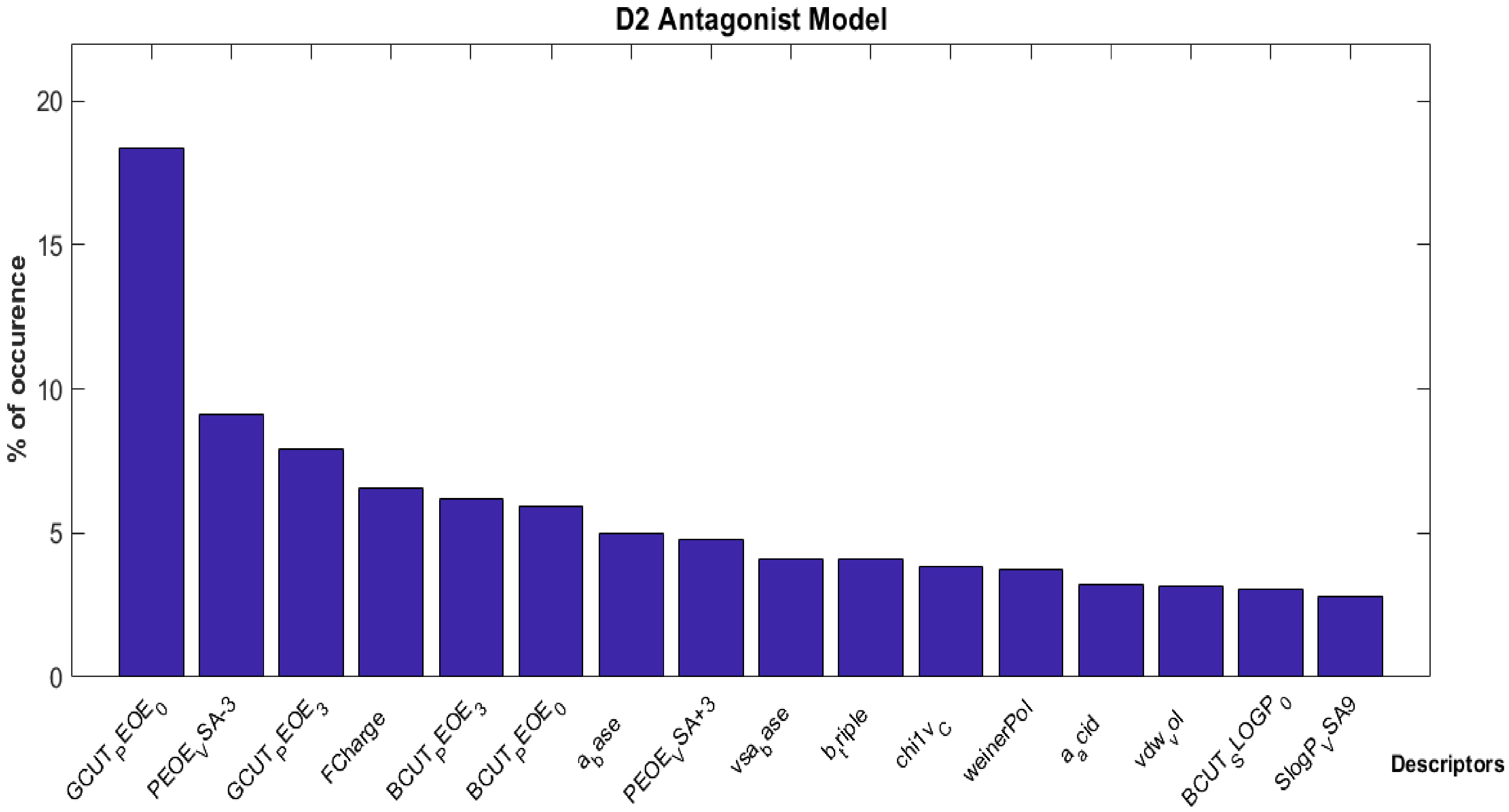

Figure S20 - Detailed 2D descriptors (%) for Dopamine D2 antagonist model

2D descriptors (MOE v.2011.10) with occurrence above 2%
