# Supplementary material for: Activity Models of Key GPCR Families in the Central Nervous System: A Tool for Many Purposes
Source: J Chem Inf Model. 2023 May 31;63(11):3248–62. doi: 10.1021/acs.jcim.2c01531 (PMC10268961; doi:10.1021/acs.jcim.2c01531)
Supplement: Supplementary file 3 — ci2c01531_si_003.pdf [file ci2c01531_si_003.pdf]

MOE 2D Molecular Descriptors

The 2011.10 release of Chemical Computing Group's Molecular Operating Environment (MOE) software.

Physical Properties

The following physical properties can be calculated from the connection table (with no dependence on conformation) of a molecule:

Code	Description
`apol`	Sum of the atomic polarizabilities (including implicit hydrogens) with polarizabilities taken from [CRC 1994].
`bpol`	Sum of the absolute value of the difference between atomic polarizabilities of all bonded atoms in the molecule (including implicit hydrogens) with polarizabilities taken from [CRC 1994].
`density`	Molecular mass density: `Weight` divided by `vdw_vol` (amu/Å^3).
`FCharge`	Total charge of the molecule (sum of formal charges).
`mr`	Molecular refractivity (including implicit hydrogens). This property is calculated from an 11 descriptor linear model [MREF 1998] with r^2 = 0.997, RMSE = 0.168 on 1,947 small molecules.
`SMR`	Molecular refractivity (including implicit hydrogens). This property is an atomic contribution model [Crippen 1999] that assumes the correct protonation state (washed structures). The model was trained on ~7000 structures and results may vary from the `mr` descriptor.
`Weight`	Molecular weight (including implicit hydrogens) in atomic mass units with atomic weights taken from [CRC 1994].
`logP(o/w)`	Log of the octanol/water partition coefficient (including implicit hydrogens). This property is calculated from a linear atom type model [LOGP 1998] with r^2 = 0.931, RMSE=0.393 on 1,827 molecules.
`logS`	Log of the aqueous solubility (mol/L). This property is calculated from an atom contribution linear atom type model [Hou 2004] with r^2 = 0.90, ~1,200 molecules.

`mutagenic`	Indicator of the presence of potentially toxic groups. A non-zero value indicates that the molecule contains a mutagenic group. The table of mutagenic groups is based on the Kazius set [Kazius 2005].
`reactive`	Indicator of the presence of reactive groups. A non-zero value indicates that the molecule contains a reactive group. The table of reactive groups is based on the Oprea set [Oprea 2000] and includes metals, phospho-, N/O/S-N/O/S single bonds, thiols, acyl halides, Michael Acceptors, azides, esters, etc.
`rsynth`	A value in [0,1] indicating the synthetic reasonableness, or feasibility, of the chemical structure. A value of 0 means it is unlikely that the molecule can be synthesized while a value of 1 means that it is likely that the molecule can be synthesized. The value reflects the fraction of heavy atoms in the molecule that can be traced back to starting materials fragments resulting from retrosynthetic disconnection rules. This molecular descriptor can also be calculated on molecules and databases using the SVL `Retrosynth*` function calls.
`SlogP`	Log of the octanol/water partition coefficient (including implicit hydrogens). This property is an atomic contribution model [Crippen 1999] that calculates logP from the given structure; i.e. the correct protonation state (washed structures). Results may vary from the `logP(o/w)` descriptor. The training set for SlogP was ~7000 structures.
`TPSA`	Polar surface area (Å^2) calculated using group contributions to approximate the polar surface area from connection table information only. The parameterization is that of Ertl *et al.* [Ertl 2000].
`vdw_vol`	van der Waals volume (Å^3) calculated using a connection table approximation.
`vdw_area`	Area of van der Waals surface (Å^2) calculated using a connection table approximation.

Subdivided Surface Areas

The Subdivided Surface Areas are descriptors based on an approximate accessible van der Waals surface area (in Å^2) calculation for each atom, v_i along with some other atomic property, p_i. The v_i are calculated using a connection table approximation. Each descriptor in a series is defined to be the sum of the v_i over all atoms i such that p_i is in a specified range (a,b).

In the descriptions to follow, L_i denotes the contribution to logP(o/w) for atom *i* as calculated in the SlogP descriptor [Crippen 1999]. R_i denotes the contribution to Molar Refractivity for atom *i* as calculated in the SMR descriptor [Crippen 1999]. The ranges were determined by percentile subdivision over a large collection of compounds.

Code	Description
SlogP_VSA0	Sum of v_i such that L_i <= -0.4.
SlogP_VSA1	Sum of v_i such that L_i is in (-0.4,-0.2].
SlogP_VSA2	Sum of v_i such that L_i is in (-0.2,0].
SlogP_VSA3	Sum of v_i such that L_i is in (0,0.1].
SlogP_VSA4	Sum of v_i such that L_i is in (0.1,0.15].
SlogP_VSA5	Sum of v_i such that L_i is in (0.15,0.20].
SlogP_VSA6	Sum of v_i such that L_i is in (0.20,0.25].
SlogP_VSA7	Sum of v_i such that L_i is in (0.25,0.30].
SlogP_VSA8	Sum of v_i such that L_i is in (0.30,0.40].
SlogP_VSA9	Sum of v_i such that L_i > 0.40.
SMR_VSA0	Sum of v_i such that R_i is in [0,0.11].
SMR_VSA1	Sum of v_i such that R_i is in (0.11,0.26].
SMR_VSA2	Sum of v_i such that R_i is in (0.26,0.35].
SMR_VSA3	Sum of v_i such that R_i is in (0.35,0.39].
SMR_VSA4	Sum of v_i such that R_i is in (0.39,0.44].
SMR_VSA5	Sum of v_i such that R_i is in (0.44,0.485].
SMR_VSA6	Sum of v_i such that R_i is in (0.485,0.56].
SMR_VSA7	Sum of v_i such that R_i > 0.56.

Atom Counts and Bond Counts

The atom count and bond count descriptors are functions of the counts of atoms and bonds (subdivided according to various criteria).

Code	Description
a_aro	Number of aromatic atoms.
a_count	Number of atoms (including implicit hydrogens). This is calculated as the sum of $(1 + h_i)$ over all non-trivial atoms i.
a_heavy	Number of heavy atoms $\#\{Z_i \mid Z_i > 1\}$.
a_ICM	Atom information content (mean). This is the entropy of the element distribution in the molecule (including implicit hydrogens but not lone pair pseudo-atoms). Let n_i be the number of occurrences of atomic number i in the molecule. Let $p_i = n_i / n$ where n is the sum of the n_i. The value of a_ICM is the negative of the sum over all i of $p_i \log p_i$.
a_IC	Atom information content (total). This is calculated to be a_ICM times n.
a_nH	Number of hydrogen atoms (including implicit hydrogens). This is calculated as the sum of h_i over all non-trivial atoms i plus the number of non-trivial hydrogen atoms.
a_nB	Number of boron atoms: $\#\{Z_i \mid Z_i = 5\}$.
a_nC	Number of carbon atoms: $\#\{Z_i \mid Z_i = 6\}$.
a_nN	Number of nitrogen atoms: $\#\{Z_i \mid Z_i = 7\}$.
a_nO	Number of oxygen atoms: $\#\{Z_i \mid Z_i = 8\}$.
a_nF	Number of fluorine atoms: $\#\{Z_i \mid Z_i = 9\}$.
a_nP	Number of phosphorus atoms: $\#\{Z_i \mid Z_i = 15\}$.
a_nS	Number of sulfur atoms: $\#\{Z_i \mid Z_i = 16\}$.
a_nCl	Number of chlorine atoms: $\#\{Z_i \mid Z_i = 17\}$.
a_nBr	Number of bromine atoms: $\#\{Z_i \mid Z_i = 35\}$.
a_nI	Number of iodine atoms: $\#\{Z_i \mid Z_i = 53\}$.
b_1rotN	Number of rotatable single bonds. Conjugated single bonds are not included (e.g. ester and peptide bonds).

b_1rotR	Fraction of rotatable single bonds: b_1rotN divided by b_heavy.
b_ar	Number of aromatic bonds.
b_count	Number of bonds (including implicit hydrogens). This is calculated as the sum of ($d_i/2 + h_i$) over all non-trivial atoms i.
b_double	Number of double bonds. Aromatic bonds are not considered to be double bonds.
b_heavy	Number of bonds between heavy atoms.
b_rotN	Number of rotatable bonds. A bond is rotatable if it has order 1, is not in a ring, and has at least two heavy neighbors.
b_rotR	Fraction of rotatable bonds: b_rotN divided by b_heavy.
b_single	Number of single bonds (including implicit hydrogens). Aromatic bonds are not considered to be single bonds.
b_triple	Number of triple bonds. Aromatic bonds are not considered to be triple bonds.
chiral	The number of chiral centers.
chiral_u	The number of unconstrained chiral centers.
lip_acc	The number of O and N atoms.
lip_don	The number of OH and NH atoms.
lip_druglike	One if and only if lip_violation < 2 otherwise zero.
lip_violation	The number of violations of Lipinski's Rule of Five [Lipinski 1997].
nmol	The number of molecules (connected components).
opr_brigid	The number of rigid bonds from [Oprea 2000].
opr_leadlike	One if and only if opr_violation < 2 otherwise zero.
opr_nring	The number of ring bonds from [Oprea 2000].
opr_nrot	The number of rotatable bonds from [Oprea 2000].

`opr_violation`	The number of violations of Oprea's lead-like test [Oprea 2000].
`rings`	The number of rings.
`VAdjMa`	Vertex adjacency information (magnitude): $1 + \log_2 m$ where *m* is the number of heavy-heavy bonds. If *m* is zero, then zero is returned.
`VAdjEq`	Vertex adjacency information (equality): $-(1-f)\log_2(1-f) - f \log_2 f$ where $f = (n^2 - m) / n^2$, *n* is the number of heavy atoms and *m* is the number of heavy-heavy bonds. If *f* is not in the open interval (0,1), then 0 is returned.

Kier & Hall Connectivity and Kappa Shape Indices

For a heavy atom *i* let $v_i = (p_i - h_i) / (Z_i - p_i - 1)$ where p_i is the number of s and p valence electrons of atom *i*. The Kier and Hall chi connectivity indices are calculated from the heavy atom degree d_i (number of heavy neighbors) and v_i. The Kier and Hall kappa molecular shape indices [Hall 1991] compare the molecular graph with minimal and maximal molecular graphs, and are intended to capture different aspects of molecular shape. In the following description, *n* denotes the number of atoms in the hydrogen suppressed graph, *m* is the number of bonds in the hydrogen suppressed graph and *a* is the sum of $(r_i/r_c - 1)$ where r_i is the covalent radius of atom *i*, and r_c is the covalent radius of a carbon atom. Also, let p_2 denote the number of paths of length 2 and p_3 the number of paths of length 3.

Code	Description
`chi0`	Atomic connectivity index (order 0) from [Hall 1991] and [Hall 1977]. This is calculated as the sum of $1/\mathrm{sqrt}(d_i)$ over all heavy atoms *i* with $d_i > 0$.
`chi0_C`	Carbon connectivity index (order 0). This is calculated as the sum of $1/\mathrm{sqrt}(d_i)$ over all carbon atoms *i* with $d_i > 0$.
`chi1`	Atomic connectivity index (order 1) from [Hall 1991] and [Hall 1977]. This is calculated as the sum of $1/\mathrm{sqrt}(d_i d_j)$ over all bonds between heavy atoms *i* and *j* where $i < j$.
`chi1_C`	Carbon connectivity index (order 1). This is calculated as the sum of $1/\mathrm{sqrt}(d_i d_j)$ over all bonds between carbon atoms *i* and *j* where $i < j$.
`chi0v`	Atomic valence connectivity index (order 0) from [Hall 1991] and [Hall 1977]. This is calculated as the sum of $1/\mathrm{sqrt}(v_i)$ over all heavy atoms *i* with $v_i > 0$.

`chi0v_C`	Carbon valence connectivity index (order 0). This is calculated as the sum of 1/sqrt(v_i) over all carbon atoms i with $v_i > 0$.
`chi1v`	Atomic valence connectivity index (order 1) from [Hall 1991] and [Hall 1977]. This is calculated as the sum of 1/sqrt($v_i v_j$) over all bonds between heavy atoms i and j where $i < j$.
`chi1v_C`	Carbon valence connectivity index (order 1). This is calculated as the sum of 1/sqrt($v_i v_j$) over all bonds between carbon atoms i and j where $i < j$.
`Kier1`	First kappa shape index: $(n\text{-}1)^2 / m^2$ [Hall 1991].
`Kier2`	Second kappa shape index: $(n\text{-}1)^2 / m^2$ [Hall 1991].
`Kier3`	Third kappa shape index: $(n\text{-}1)(n\text{-}3)^2 / p_3^2$ for odd n, and $(n\text{-}3)(n\text{-}2)^2 / p_3^2$ for even n [Hall 1991].
`KierA1`	First alpha modified shape index: $s(s\text{-}1)^2 / m^2$ where $s = n + a$ [Hall 1991].
`KierA2`	Second alpha modified shape index: $s(s\text{-}1)^2 / m^2$ where $s = n + a$ [Hall 1991].
`KierA3`	Third alpha modified shape index: $(s\text{-}1)(s\text{-}3)^2 / p_3^2$ for odd n, and $(s\text{-}3)(s\text{-}2)^2 / p_3^2$ for even n where $s = n + a$ [Hall 1991].
`KierFlex`	Kier molecular flexibility index: (`KierA1`) (`KierA2`) / n [Hall 1991].
`zagreb`	Zagreb index: the sum of d_i^2 over all heavy atoms i.

Adjacency and Distance Matrix Descriptors

The *adjacency matrix*, M, of a chemical structure is defined by the elements [M_{ij}] where M_{ij} is 1 if atoms i and j are bonded and zero otherwise. The *distance matrix*, D, of a chemical structure is defined by the elements [D_{ij}] where D_{ij} is the length of the shortest path from atoms i to j; zero is used if atoms i and j are not part of the same connected component. The adjacency matrix of CH3CH=O is displayed on the left and its distance matrix is displayed on the right (below):

```
C1      0 1 1 1 1 0 0      0 1 1 1 1 2 2
H2      1 0 0 0 0 0 0      1 0 2 2 2 3 3
H3      1 0 0 0 0 0 0      1 2 0 2 2 3 3
H4      1 0 0 0 0 0 0      1 2 2 0 2 3 3
C5      1 0 0 0 0 1 1      1 2 2 2 0 1 1
H6      0 0 0 0 1 0 0      2 3 3 3 1 0 2
O7      0 0 0 0 1 0 0      2 3 3 3 1 2 0
```

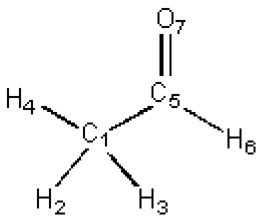

Petitjean [Petitjean 1992] defines the **eccentricity** of a vertex to be the longest path from that vertex to any other vertex in the graph. The graph **radius** is the smallest vertex eccentricity in the graph and the graph **diameter** as the largest vertex eccentricity. These values are calculated using the distance matrix and are used for several descriptors described below.

The following descriptors are calculated from the distance and adjacency matrices of the heavy atoms:

Code	Description
`balabanJ`	Balaban's connectivity topological index [Balaban 1982].
`BCUT_PEOE_0`<br>`BCUT_PEOE_1`<br>`BCUT_PEOE_2`<br>`BCUT_PEOE_3`	The BCUT descriptors [Pearlman 1998] are calculated from the eigenvalues of a modified adjacency matrix.<br>Each *ij* entry of the adjacency matrix takes the value 1/sqrt(b_{ij}) where b_{ij} is the formal bond order between bonded atoms *i* and *j*. The diagonal takes the value of the PEOE partial charges. The resulting eigenvalues are sorted and the smallest, 1/3-ile, 2/3-ile and largest eigenvalues are reported.
`BCUT_SLOGP_0`<br>`BCUT_SLOGP_1`<br>`BCUT_SLOGP_2`<br>`BCUT_SLOGP_3`	The BCUT descriptors using atomic contribution to logP (using the Wildman and Crippen SlogP method) instead of partial charge.
`BCUT_SMR_0`<br>`BCUT_SMR_1`<br>`BCUT_SMR_2`<br>`BCUT_SMR_3`	The BCUT descriptors using atomic contribution to molar refractivity (using the Wildman and Crippen SMR method) instead of partial charge.
`diameter`	Largest value in the distance matrix [Petitjean 1992].
`petitjean`	Value of (`diameter` - `radius`) / `diameter`.
`GCUT_PEOE_0`<br>`GCUT_PEOE_1`<br>`GCUT_PEOE_2`<br>`GCUT_PEOE_3`	The GCUT descriptors are calculated from the eigenvalues of a modified graph distance adjacency matrix. Each *ij* entry of the adjacency matrix takes the value 1/sqr(d_{ij}) where d_{ij} is the (modified) graph distance between atoms *i* and *j*. The diagonal takes the value of the PEOE partial charges. The resulting eigenvalues are sorted and the smallest, 1/3-ile, 2/3-ile and largest eigenvalues are reported.
`GCUT_SLOGP_0`<br>`GCUT_SLOGP_1`<br>`GCUT_SLOGP_2`<br>`GCUT_SLOGP_3`	The GCUT descriptors using atomic contribution to logP (using the Wildman and Crippen SlogP method) instead of partial charge.
`GCUT_SMR_0`<br>`GCUT_SMR_1`	The GCUT descriptors using atomic contribution to molar refractivity (using the Wildman and Crippen SMR method) instead of partial charge.

GCUT_SMR_2<br>GCUT_SMR_3	
petitjeanSC	Petitjean graph Shape Coefficient as defined in [Petitjean 1992]: (diameter - radius) / radius.
radius	If r_i is the largest matrix entry in row i of the distance matrix D, then the radius is defined as the smallest of the r_i [Petitjean 1992].
VDistEq	If m is the sum of the distance matrix entries then VdistEq is defined to be the sum of $\log_2 m - p_i \log_2 p_i / m$ where p_i is the number of distance matrix entries equal to i.
VDistMa	If m is the sum of the distance matrix entries then VDistMa is defined to be the sum of $\log_2 m - D_{ij} \log_2 D_{ij} / m$ over all i and j.
wienerPath	Wiener path number: half the sum of all the distance matrix entries as defined in [Balaban 1979] and [Wiener 1947].
wienerPol	Wiener polarity number: half the sum of all the distance matrix entries with a value of 3 as defined in [Balaban 1979].

Pharmacophore Feature Descriptors

The Pharmacophore Atom Type descriptors consider only the heavy atoms of a molecule and assign a type to each atom. That is, hydrogens are suppressed during the calculation. The atom typing mechanism is located in the file `$MOE/lib/svl/ph4.svl/ph4type.svl` which is a rule-based system for assigning pharmacophore features to atoms. The feature set is Donor, Acceptor, Polar (both Donor and Acceptor), Positive (base), Negative (acid), Hydrophobe and Other. Assignments may take into account implied protonation, deprotonation, keto/enol considerations and tautomerism at a biologically relevant pH. For example, -COOH will be typed in its deprotonated form regardless of how the structure is stored.

Code	Description
a_acc	Number of hydrogen bond acceptor atoms (not counting acidic atoms but counting atoms that are both hydrogen bond donors and acceptors such as -OH).
a_acid	Number of acidic atoms.
a_base	Number of basic atoms.

a_don	Number of hydrogen bond donor atoms (not counting basic atoms but counting atoms that are both hydrogen bond donors and acceptors such as -OH).
a_hyd	Number of hydrophobic atoms.
vsa_acc	Approximation to the sum of VDW surface areas ($Å^2$) of pure hydrogen bond acceptors (not counting acidic atoms and atoms that are both hydrogen bond donors and acceptors such as -OH).
vsa_acid	Approximation to the sum of VDW surface areas of acidic atoms ($Å^2$).
vsa_base	Approximation to the sum of VDW surface areas of basic atoms ($Å^2$).
vsa_don	Approximation to the sum of VDW surface areas of pure hydrogen bond donors (not counting basic atoms and atoms that are both hydrogen bond donors and acceptors such as -OH) ($Å^2$).
vsa_hyd	Approximation to the sum of VDW surface areas of hydrophobic atoms ($Å^2$).
vsa_other	Approximation to the sum of VDW surface areas ($Å^2$) of atoms typed as "other".
vsa_pol	Approximation to the sum of VDW surface areas ($Å^2$) of polar atoms (atoms that are both hydrogen bond donors and acceptors), such as -OH.

Partial Charge Descriptors

Descriptors that depend on the partial charge of each atom of a chemical structure require calculation of those partial charges. An unfortunate complication is the fact that there are numerous methods of calculating partial charges. Rather than enforce a particular method, MOE provides several versions of most of the charge-dependent descriptors. The only difference between these variants is the source of the partial charges. The following variants are supported: PEOE, Q (described below).

PEOE. The Partial Equalization of Orbital Electronegativities (PEOE) method of calculating atomic partial charges [Gasteiger 1980] is a method in which charge is transferred between bonded atoms until equilibrium. To guarantee convergence, the amount of charge transferred at each iteration is damped with an exponentially decreasing scale factor. The amount of charge transferred, dq_{ij}, between atoms i and j when $X_i > X_j$ is

$$dq_{ij} = (1/2^k)\ (X_i - X_j) / X_j^+$$

where X_j^+ is the electronegativity of the positive ion of atom j; X_i is the electronegativity of atom i (quadratically dependent on partial charge); and k is the iteration number of the algorithm. Electronegativity values are determined by parameterization found in the SVL

source code file`$MOE/lib/svl/calc.svl/charge.svl`. The PEOE charges depend only on the connectivity of the input structures: elements, formal charges and bond orders. Descriptors using the PEOE charges are prefixed with `PEOE_`.

Q. Descriptors prefixed with `Q_` use the partial charges stored with each structure in the database. In other words, no partial charge calculation is made and it is assumed that some external program has been used to calculate the atomic partial charges. This dependence can be a subtle source of error if, for example, the wrong charges are stored when descriptors are recalculated (e.g. when evaluating QSAR models on novel structures).

Partial charges from forcefields can be used by energy minimizing the database structures (which will store the charges), then using the `Q_` variant of the descriptors.

Warning! Most partial charge methods require that all hydrogens be explicit in the stored structures. This can be a source of error when recalculating descriptors on novel structures during QSAR model evaluation.

Let q_i denote the partial charge of atom *i* as defined above. Let v_i be the van der Waals surface area ($Å^2$) of atom *i* (as calculated by a connection table approximation). The following descriptors are calculated:

Code	Description
`Q_PC+`<br>`PEOE_PC+`	Total positive partial charge: the sum of the positive q_i. `Q_PC+` is identical to `PC+` which has been retained for compatibility.
`Q_PC-`<br>`PEOE_PC-`	Total negative partial charge: the sum of the negative q_i. `Q_PC-` is identical to `PC-` which has been retained for compatibility.
`Q_RPC+`<br>`PEOE_RPC+`	Relative positive partial charge: the largest positive q_i divided by the sum of the positive q_i. `Q_RPC+` is identical to `RPC+` which has been retained for compatibility.
`Q_PRC-`<br>`PEOE_RPC-`	Relative negative partial charge: the smallest negative q_i divided by the sum of the negative q_i. `Q_RPC-` is identical to `RPC-` which has been retained for compatibility.
`Q_VSA_POS`<br>`PEOE_VSA_POS`	Total positive van der Waals surface area. This is the sum of the v_i such that q_i is non-negative. The v_i are calculated using a connection table approximation.
`Q_VSA_NEG`<br>`PEOE_VSA_NEG`	Total negative van der Waals surface area. This is the sum of the v_i such that q_i is negative. The v_i are calculated using a connection table approximation.

Q_VSA_PPOS<br>PEOE_VSA_PPOS	Total positive polar van der Waals surface area. This is the sum of the v_i such that q_i is greater than 0.2. The v_i are calculated using a connection table approximation.
Q_VSA_PNEG<br>PEOE_VSA_PNEG	Total negative polar van der Waals surface area. This is the sum of the v_i such that q_i is less than -0.2. The v_i are calculated using a connection table approximation.
Q_VSA_HYD<br>PEOE_VSA_HYD	Total hydrophobic van der Waals surface area. This is the sum of the v_i such that $\|q_i\|$ is less than or equal to 0.2. The v_i are calculated using a connection table approximation.
Q_VSA_POL<br>PEOE_VSA_POL	Total polar van der Waals surface area. This is the sum of the v_i such that $\|q_i\|$ is greater than 0.2. The v_i are calculated using a connection table approximation.
Q_VSA_FPOS<br>PEOE_VSA_FPOS	Fractional positive van der Waals surface area. This is the sum of the v_i such that q_i is non-negative divided by the total surface area. The v_i are calculated using a connection table approximation.
Q_VSA_FNEG<br>PEOE_VSA_FNEG	Fractional negative van der Waals surface area. This is the sum of the v_i such that q_i is negative divided by the total surface area. The v_i are calculated using a connection table approximation.
Q_VSA_FPPOS<br>PEOE_VSA_FPPOS	Fractional positive polar van der Waals surface area. This is the sum of the v_i such that q_i is greater than 0.2 divided by the total surface area. The v_i are calculated using a connection table approximation.
Q_VSA_FPNEG<br>PEOE_VSA_FPNEG	Fractional negative polar van der Waals surface area. This is the sum of the v_i such that q_i is less than -0.2 divided by the total surface area. The v_i are calculated using a connection table approximation.
Q_VSA_FHYD<br>PEOE_VSA_FHYD	Fractional hydrophobic van der Waals surface area. This is the sum of the v_i such that $\|q_i\|$ is less than or equal to 0.2 divided by the total surface area. The v_i are calculated using a connection table approximation.
Q_VSA_FPOL<br>PEOE_VSA_FPOL	Fractional polar van der Waals surface area. This is the sum of the v_i such that $\|q_i\|$ is greater than 0.2 divided by the total surface area. The v_i are calculated using a connection table approximation.
PEOE_VSA+6	Sum of v_i where q_i is greater than 0.3.

PEOE_VSA+5	Sum of v_i where q_i is in the range [0.25,0.30).
PEOE_VSA+4	Sum of v_i where q_i is in the range [0.20,0.25).
PEOE_VSA+3	Sum of v_i where q_i is in the range [0.15,0.20).
PEOE_VSA+2	Sum of v_i where q_i is in the range [0.10,0.15).
PEOE_VSA+1	Sum of v_i where q_i is in the range [0.05,0.10).
PEOE_VSA+0	Sum of v_i where q_i is in the range [0.00,0.05).
PEOE_VSA-0	Sum of v_i where q_i is in the range [-0.05,0.00).
PEOE_VSA-1	Sum of v_i where q_i is in the range [-0.10,-0.05).
PEOE_VSA-2	Sum of v_i where q_i is in the range [-0.15,-0.10).
PEOE_VSA-3	Sum of v_i where q_i is in the range [-0.20,-0.15).
PEOE_VSA-4	Sum of v_i where q_i is in the range [-0.25,-0.20).
PEOE_VSA-5	Sum of v_i where q_i is in the range [-0.30,-0.25).
PEOE_VSA-6	Sum of v_i where q_i is less than -0.30.
